# Prevalence and determinants of return to work after various coronary events: meta-analysis of prospective studies

**DOI:** 10.1038/s41598-022-19467-z

**Published:** 2022-09-12

**Authors:** Samantha Huo Yung Kai, Jean Ferrières, Mélisande Rossignol, Frédéric Bouisset, Julie Herry, Yolande Esquirol

**Affiliations:** 1 CERPOP, Université de Toulouse, Inserm, UPS, 31000 Toulouse, France; 2 Department of Epidemiology, Toulouse University Hospital, 31000 Toulouse, France; 3 Department of Cardiology, Toulouse University Hospital, 31400 Toulouse, France; 4 Occupational Health Department, Toulouse University Hospital, 31300 Toulouse, France

**Keywords:** Occupational health, Public health, Epidemiology, Cardiovascular diseases, Health care

## Abstract

Return to work (RTW) after a coronary event remains a major concern. This systematic review and meta-analysis of prospective studies published between January 1988 and August 2020, aim to evaluate the prevalence of RTW after a coronary event (myocardial infarction, acute coronary syndrome, angina pectoris) and to assess the determinants of RTW (such as follow-up duration, date of recruitment, country, gender, occupational factors, etc.). PRISMA and MOOSE guidelines were followed. Study quality was assessed using the Newcastle–Ottawa Scale. Random-effects models were carried out to determine pooled prevalence estimates and 95% confident interval. A total of 43 prospective studies (34,964 patients) were investigated. RTW overall random effects pooled prevalence was estimated at 81.1% [95% CI 75.8–85.8]. Country, year of implementation or gender did not significantly modify the prevalence estimates. Lower level of education and degraded left ventricular ejection fraction decreased RTW prevalence estimates (respectively, 76.1% vs 85.6% and 65.3% vs 77.8%). RTW prevalence estimates were higher for white-collars (81.2% vs 65.0% for blue-collars) and people with low physical workload (78.3% vs 64.1% for elevated physical workload).Occupational physical constraints seem to have a negative role in RTW while psycho-logical factors at work are insufficiently investigated. A better understanding of the real-life working conditions influencing RTW would be useful to maintain coronary patients in the labor market.

## Introduction

Major progress in the diagnosis and in the treatment of acute coronary syndromes (ACS) have been made in the last decades and a decrease of mortality have been reported in several countries^1^. Indeed, transluminal angioplasty introduced in 1977 by Grüntzig, treatment with coronary stents 10 years later, and the introduction of the aspirin-clopidogrel association and drug-eluting stents in 1999 have considerably improved the prognosis of these patients. Cardiac rehabilitation programs have also demonstrated their effectiveness in reducing rehospitalizations and cardiovascular mortality^2^ but are still unequally available^3,4^. Despite better patient care and prevention, ischemic heart disease remains the first cause of death with 16% of the world total deaths in 2019 (WHO: https://www.who.int/news-room/fact-sheets/detail/the-top-10-causes-of-death), with an increase of disability-adjusted life years and years of life lost which doubled between 1990 and 2019^5^. Thus, complications in surviving patients could delay or prevent return to work (RTW). Furthermore, as duration of work life rises in most of the countries and ACS frequency increases with age, more people with ACS remain in labor market.

Data from retrospective studies indicate that the proportion of RTW 1 year after a coronary event varies in a large range, between 60 and 90%^6–8^. Moreover, these studies highlight that a substantial number of patients who returned to work after an ACS do not maintain their occupational activities 1 year later^8,9^, resulting in a high economic burden for both patients and society^10^.While a successful RTW after an ACS depending on medical, psychological, and occupational optimized management could make sense, trials have not provided compelling proofs of the effectiveness of several type of interventions on RTW^11^, thus questioning the type of the parameters influencing RTW. For instance, RTW between 6 and 12 months after the events was not improved with psychological interventions (RR = 1.24 [95%: CI 0.95; 1.63]) or physical rehabilitation (RR = 1.09 [95%: CI 0.99; 1.20]), and work-directed counselling did not reduce time to RTW^11^. More recently, a meta-analysis of seven controlled trials focusing on the cardiac rehabilitation effect shows a slight difference in the prevalence of RTW between patients with or without cardiac rehabilitation (respectively, 62% and 58%)^12^.

However, numerous prospective studies were conducted in the last 40 years, providing potentially robust data to establish a prevalence estimate of RTW and to assess the factors influencing RTW, which could be of use to improve patients care and optimize work resumption. Therefore, the primary objective of this systematic review and meta-analysis, based on prospective studies, is to estimate the prevalence of RTW after various types of coronary events (myocardial infarction—MI, ACS, angina pectoris). The secondary objective is to study the determinants of RTW (follow-up duration, date of recruitment, country of the study, study quality, gender, left ventricular ejection fraction (LVEF), educational level, occupational factors, treatment– percutaneous coronary transluminal angioplasty, PCTA or coronary artery bypass graft surgery, CABG).

## Materials and methods

This systematic review was registered in the prospective register of systematic reviews (PROSPERO, April 2020, registration number CRD42020152600). We used PRISMA guidelines^13^ and MOOSE (Meta-analyses Of Observational Studies in Epidemiology) checklist^14^ to define the protocol and report this systematic review.

### Search strategy

A literature research in MEDLINE (PubMed), Web of Science, and Cochrane library databases was conducted to identify the studies published from January 1st, 1988 (marking the year when Smith and O’Rourke published the first study putting forward some explicative hypotheses on the consequences of MI on work resumption with the need to clarify them in the upcoming years^15^) to the August 31st, 2020.

The following MeSH terms and keywords were searched in titles, abstracts, and full texts:

- Mesh terms:
  - myocardial infarction, ACS, angina pectoris, angioplasties, coronary artery bypass,
  - return to work, sick leave.
- Keywords:
  - myocardial infarction, heart attack, angina, coronary syndrome,
  - return to work, return to occupational activity, work resumption, sickness absence.

### Study eligibility criteria

All observational prospective studies assessing RTW in adults after a cardiovascular event (MI, ACS, and angina pectoris) were included. Retained studies were written in English or in French. Retrospective, cross-sectional studies, controlled trials, case reports, oral presentations, posters, opinion articles, books, and articles with only an abstract were excluded.

### Study selection

Article selection was carried out independently by three medical physicians (MR, SHYK, YE). Article list was managed with Endnote software (version X.8) and duplicates were removed using this reference manager. Screening of titles and then of abstracts was conducted by the three reviewers who also pursue a further selection after reading the full articles and checked the appropriateness of the studies. In case of disagreement, a discussion with a fourth reviewer (JF) resolved it. The references listed in the included articles were checked and compared to those obtained after the selection to ensure that important articles were not omitted.

### Data extraction and coding

Three review authors extracted and organized in a Microsoft Office Excel database the following information: first author, publication year, country, study design, recruitment period and site, study size, age, gender, type of cardiovascular events, treatment type (PCTA, CABG), occupational characteristics, number of workers returning to work (frequency and/or percentage), duration of follow-up and time to RTW (mean, median, standard deviation).

Events were classified into three categories: acute events, stable angina or elective interventions, and acute events or stable angina or elective interventions (when events were not distinguished by the authors). Patients’ follow-up duration was categorized into: ≤ 3, ]3–6], ]6–12], and > 12 months. To consider patient’s care progress, notably with the introduction of the aspirin-clopidogrel association and drug-eluting stents around 2000, recruitment time was dichotomized into two periods: before and after 2000. Study countries was organized according to the WHO (World Health Organization) regions. The method of assessment of the cardiovascular events was recoded into three classes: clinical examination from experts, patient’s interview, and ICD-10 codes obtained from registries. RTW could be self-declared or determined using administrative or employer databases or data from occupational medicine visits.

Regarding work conditions, several models have been constituted: firstly, white-collar (aggregating non-manual, executive, mostly mental workload, very light/light work) versus blue-collars workers (aggregating manual, workers, mostly physical workload, moderate/heavy work), secondly workstation with low or high occupational physical activity (OPA). Study population mean age was used to classify studies in three age categories: mean age < 51 years old, mean age between 51 and 53 years old and mean age ≥ 54 years old. Two levels of education were compared: < high school versus ≥ high school, as well as two levels of LVEF: < 40% versus ≥ 40%.

### Study quality assessment—risk of bias

We used the Newcastle–Ottawa Scale (NOS) to evaluate the study quality. This scale assesses three dimensions: selection, comparability, and outcome. Stars are assigned according to the study quality and bias risk with a maximal number of 9 stars. The higher the number of stars, the lower the bias risk is. The threshold of 7 stars at the NOS scale was used to highlight the high-quality studies.

### Data synthesis and analysis

The package “meta” (v.4.13-0) of R (v.4.0.2) and RStudio (v.1.3.1073) were used to undertake the meta-analysis. Assuming estimates may vary across studies due to differences in population or study settings (country, size, date) for instance, random-effects meta-analyses were carried out to pool the data. We used the Freeman-Tukey double arcsine transformation to include studies with extreme prevalence^16,17^. Pooled prevalence estimates of RTW along with 95% confidence interval were calculated. Studies weights were calculated using the inverse variance method. The DerSimonian and Laird method was used to estimate the between‐study variance. Heterogeneity was reported as I^2^ statistics^18^. Prediction intervals (PI) were used to describe the range of expected estimates (narrow PI indicate consistence across studies whereas wide PI indicate variation across studies)^19^. Significance threshold was set at 0.05. Subgroup analyses were conducted. To further explore heterogeneity, sensitivity analyses were undertaken to measure the effect of the size of the studies by removing some studies. Small study effects bias were assessed with funnel plot^20^ and Begg and Egger tests, and were considered significant if p-value < 0.05.

## Results

### Study characteristics

**Figure 1:**
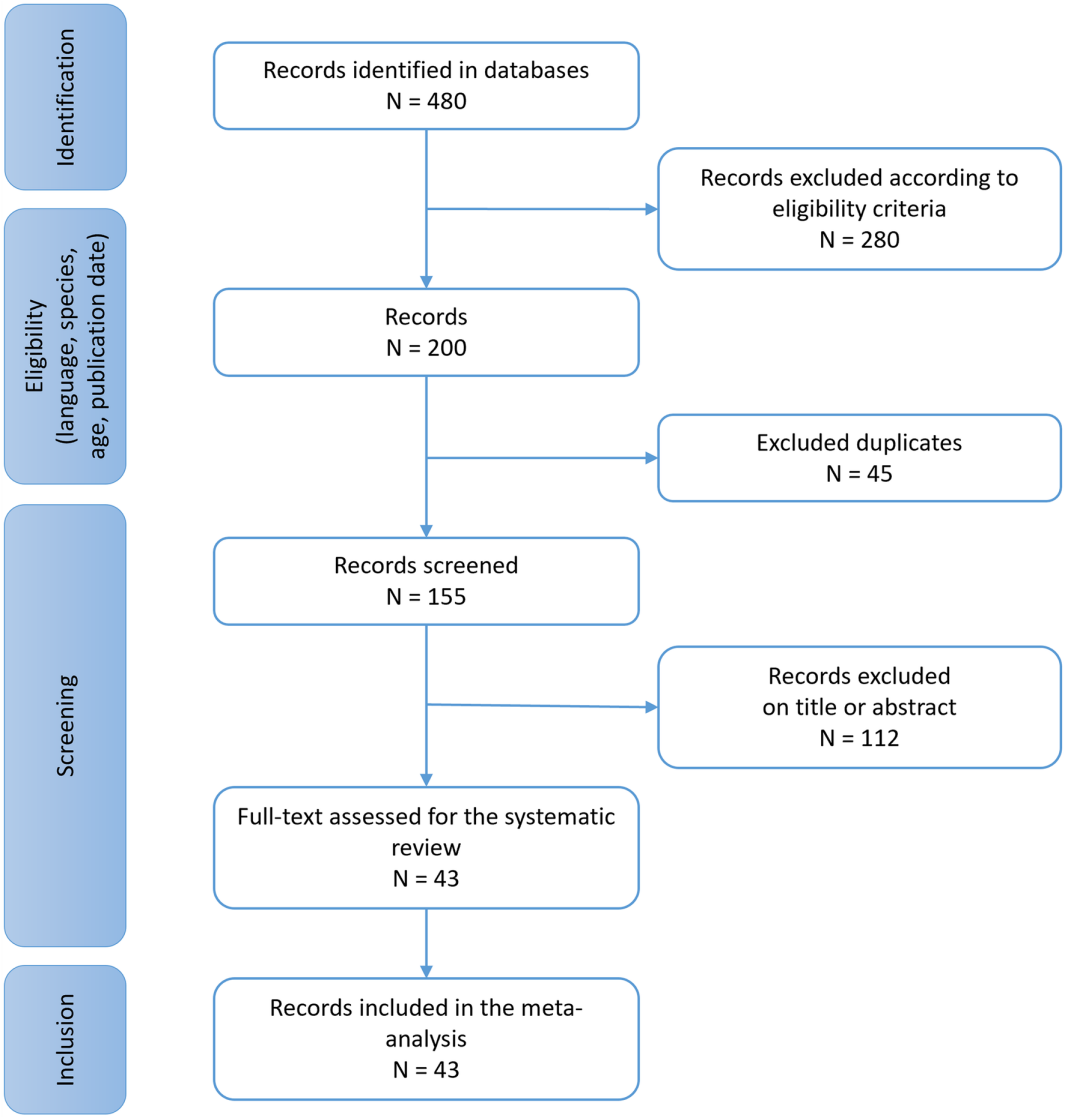
Study selection.

A total of 480 records were identified and 43 prospective studies described in Supplementary Table S1 online^21–63^ were finally included (Fig. 1). Publication years and recruitment periods range respectively from 1988 to 2018 and from 1981 to 2017. Most of the studies took place in Europe (72%) and the patients were recruited during hospital stays (91%) (Supplementary Table S2 online).

Quality and risk of bias assessment of the 43 included studies by using the NOS scale (Supplementary Fig. 1 online) reported a low risk of bias for 27 studies (7–9 stars) and a higher risk of bias for 16 studies (4–6 stars). For these last ones, the limited number of adjustment factors lead to a low score for the comparability criteria and most of them were implemented before 2010.

### RTW after a coronary event

Considering the longest follow-up time for all the studies^21–63^, RTW overall random effects pooled prevalence was calculated at 81.1% [75.8; 85.8], with substantial heterogeneity (I^2^ = 99.1% and an expected prevalence ranging between 41.2% and 100%) (Supplementary Fig. S2 online) and no evidence for small study effects (p-value respectively for Begg’s and Egger’s test: 0.267 and 0.284) (Supplementary Fig. S3 online).

**Figure 2:**
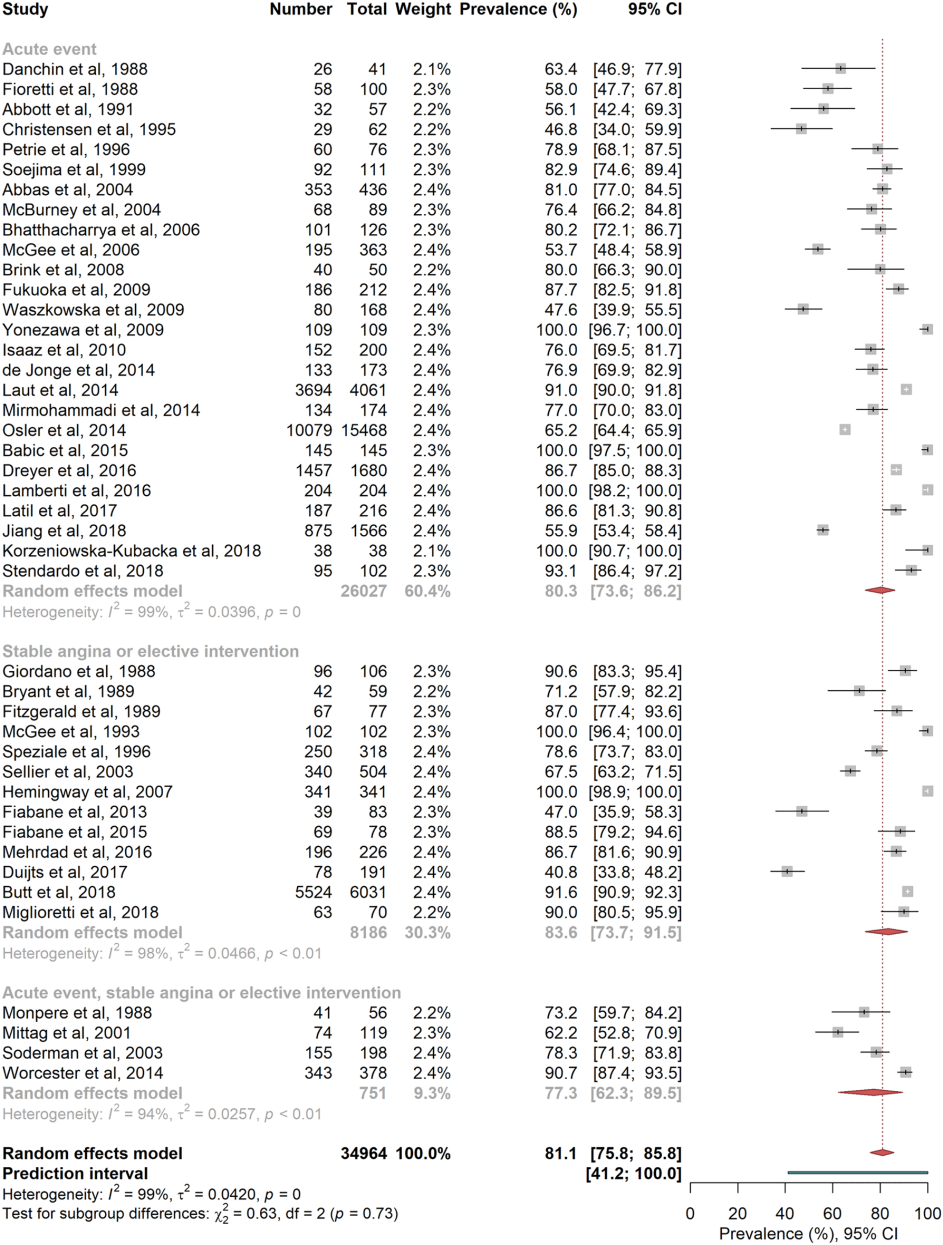
Random-effects meta-analysis of return-to-work prevalence according to the type of events. The squares and horizontal lines correspond to the study-specific prevalence and 95% CIs. Proportionally sized boxes represent the weight of each study. The diamond represents the pooled prevalence and 95% CI of the overall population. The horizontal thick line corresponds to the 95% prediction interval.

Figure 2 shows the pooled prevalence estimates of RTW according to the type of coronary events: 80.3% [73.6; 86.2] for acute events, 83.6% [73.7; 91.5] for stable angina or elective interventions, and 77.4% [62.3; 89.5] when the event was not clearly defined in the published studies (acute events or stable angina or elective interventions) (P = 0.731, I^2^ = 98.8%, no evidence for small study effects).

### RTW according to the characteristics of the studies

**Figure 3:**
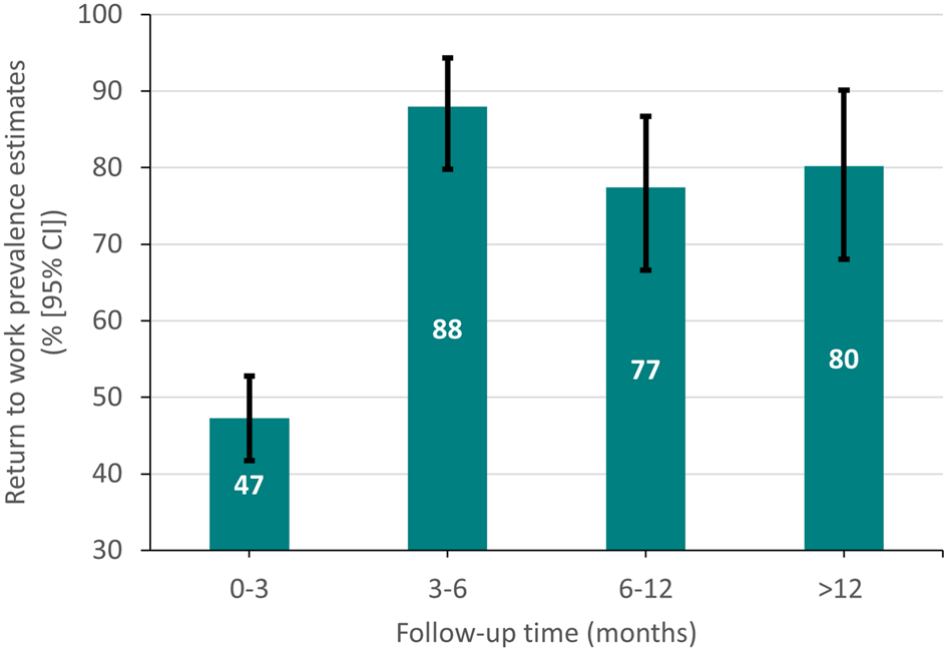
Return-to-work prevalence according to follow-up time.

RTW pooled prevalence estimates after a coronary event were significantly different (P < 0.001, I^2^ = 99.7%, PI [23.2%; 100%]) according to the follow-up periods expressed in months: ≤ 3^22,25–27,36,46–49,55,58,60,62^, ]3–6]^23,26,27,30,36,41,42,44,53,62,63^, ]6–12]^24,25,27,32–35,37–40,46–50,52,55,56,58,59^ and > 12^21,28,29,31,45,47,49,51,54,59^. Patients were more likely to return to work after 3 months following the coronary event: 47.3% [41.7; 52.8] (PI [26.6; 68.4]) of the patients had returned to work before 3 months, 88% [79.9; 94.3] (PI [50.1; 100]) between 3 and 6 months. The overall random effects pooled prevalence decreased to reach 77.4% [66.6; 86.7] (PI [20.2; 100]) between 6 and 12 months and 80.2% [68.0; 90.1%] (PI [29.3; 100]) after 12 months (Fig. 3 and Supplementary Fig. S4 online). Heterogeneity significantly decreases in the 0–3 months follow-up group when the two larger studies were removed from the model^47,49^ (I^2^ = 68.1%). There was no evidence for small study effects except for the 6–12 months follow-up subgroup (Egger test P = 0.03).

RTW pooled prevalence estimates were not significantly different between studies according to the date of recruitment (Table 1) (P = 0.458, I^2^ = 99.1%, PI [41.2%; 100%]): 79.6% [74.4; 84.4] before 2001^21–37,40,45–47,59^ (respectively P-values for Begg and Egger tests, 0.592 and 0.004) and 82.7% [75.7; 88.8] after 2001^38,39,41–44,48–58,60–63^ (respectively P-values for Begg and Egger tests, 0.809 and 0.018) (Supplementary Fig. S5 online).

**Table 1:** Return to work overall random effects pooled prevalence, according to the characteristics of the studies and the clinical and socio-professional characteristics of the patients.

		No.	RTW % [95%-CI]	I^2^	p^a^
Recruitment date	< 2001	22	79.6 [74.4; 84.4]	97.2	0.458
	≥ 2001	21	82.7 [75.7; 88.8]	98.8	
Disease definition	Clinical	37	81.3 [75.8; 86.2]	97.9	0.971
	ICD	4	78.8 [57.5; 94.0]	99.8	
	Interview	2	80.5 [74.9; 85.5]	99.7	
Outcome definition	Self-reported	36	83.9 [70.0; 94.0]	96.7	0.610
	Not self-reported	7	81.6 [75.2; 87.2]	99.8	
NOS-scale score	7 stars or more	27	80.2 [67.3; 90.6]	99.3	0.821
	< 7 stars	16	85.2 [81.1; 88.9]	97.9	
WHO region	International	3	79.8 [73.0; 85.8]	78.6	0.654
	Europe	31	85.0 [62.3; 98.4]	99.2	
	Western Pacific	5	81.9 [70.5; 91.0]	98.9	
	America	2	82.2 [71.8; 90.7]	67.0	
	Eastern Mediterranean	2	75.9 [59.6; 89.1]	84.2	
Gender	Male	11	64.4 [44.3; 82.3]	99.6	0.336
	Female	11	74.9 [62.8; 85.4]	99.0	
Age	< 51 years	6	82.0 [63.5; 95.0]	96.6	0.387
	51–53 years	9	86.2 [73.4; 95.4]	99.1	
	54 years and more	7	65.3 [45.2; 83.0]	98.2	
Education level	< High school	7	85.6 [81.2; 89.6]	87.6	0.085
	≥ High school	7	80.8 [67.1; 91.5]	74.2	
LVEF	< 40%	5	77.8 [57.1; 93.2]	86.4	0.346
	≥ 40%	5	76.1 [64.1; 86.5]	98.2	
Treatment	PCTA	11	75.8 [65.9; 84.5]	99.0	0.524
	CABG	11	80.5 [74.9; 85.5]	96.9	

CABG, coronary artery bypass graft surgery; CI, confidence interval; ICD, International Classification of Diseases; LVEF, Left Ventricular Ejection Fraction;N°, number of studies; NOS, Newcastle–Ottawa scale; P, p-value; PCTA, percutaneous coronary transluminal angioplasty; RTW %, return to work overall random effects pooled prevalence.

^a^Between group difference.

To assess the impact of the method used to characterize the coronary event on RTW, three subgroups were identified: studies using clinical diagnosis established by experts^21–34,36,38,39,41–48,50–53,55–58,60–63^, studies collecting the information (ICD) in population register databases^35,37,49,59^, and studies based on patient interviews^40,54^. Whatever the subgroups, no significant difference (P = 0.971, I^2^ = 99.1%, PI [41.2%; 100%], no evidence for small study effects) was observed although pooled prevalence estimate calculated for studies using register databases was lower (78.8%) compared to the others (81.3% with clinical diagnosis and 81.1% with interviews) (Table 1 and Supplementary Fig. S6 online).

To analyze the effect of the method to measure RTW, studies were dichotomized (Table 1 and Supplementary Fig. S7 online): studies based on self-report^21–23,25–39,41–46,48,50–54,57,58,60–63^ and studies using administrative or employer databases or occupational medicine consultation^24,40,47,49,55,56,59^, and no significant difference was noticed (respectively, 80.5% and 83.9%, P = 0.610, I^2^ = 99.1%, PI [41.2%; 100%], no evidence for small study effects).

The pooled prevalence estimates did not differ according to study quality defined with the NOS scale^21–63^: 81.6% for score ≥ 7 and 80.2% for score < 7 (P = 0.821, I^2^ = 99.1%, PI [41.2%; 100%], no evidence for small study effects) (Table 1 and Supplementary Fig. S8 online).

The region^21–63^ where the studies took place did not impact RTW pooled prevalence estimates (P = 0.654, I^2^ = 99.1%, PI [41.2%; 100%], no evidence for small study effects) (Table 1 and Supplementary Fig. S9 online).

### RTW according to patients clinical and socio-professional characteristics

Overall random effects pooled prevalence was higher in men than in women (75.9% vs. 64.4%) but the difference was not significant (P = 0.336, I^2^ = 99.4%, PI [22.5%; 99.9%]) (Table 1 and Supplementary Fig. S10 online). Small study effects were observed for the men subgroup (respectively P-values for Begg and Egger tests, 0.938 and 0.001).

People aged less than 51 years old tended to present lower RTW prevalence estimates (74.9%), compared to the 51–53 years old (82%), and people aged 54 years old or older (86.2%) (P = 0.387, I^2^ = 98.8%, PI [37.6%; 100%], no evidence for small study effects) (Table 1 and Supplementary Fig. S11 online).

RTW prevalence estimates did not significantly differ according to LVEF^21,46,48,50,56^ (respectively 65.3% and 77.8% for < 40% and ≥ 40%, P = 0.346, I^2^ = 96.5%, PI [19.3%; 100%]) (Table 1 and Supplementary Fig. S12 online).

Education level was not significantly associated to RTW^26,35,42,46,50,52,63^ (respectively 76.1% and 85.6% for < high school and ≥ high school, P = 0.085, I^2^ = 82.9%, PI [62.8%; 95.5%], no evidence for small study effects) (Table 1 and Supplementary Fig. S13 online).

**Figure 4:**
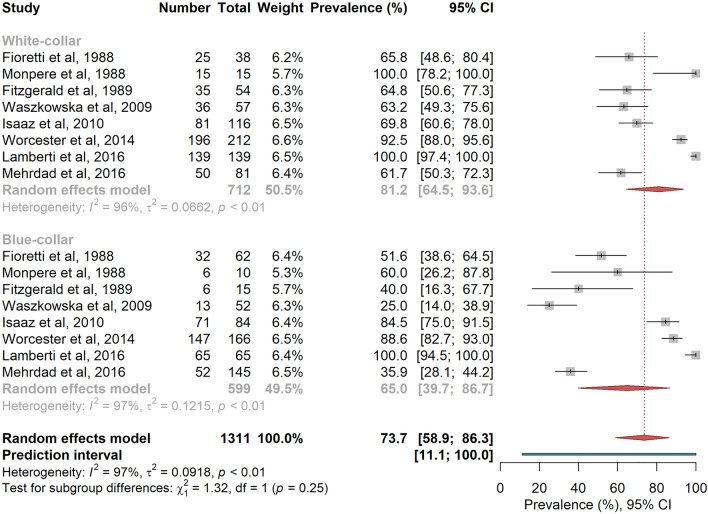
Random-effects meta-analysis of return-to-work prevalence according to socio-professional category. The squares and horizontal lines correspond to the study-specific prevalence and 95% CIs. Proportionally sized boxes represent the weight of each study. The diamond represents the pooled prevalence and 95% CI of the overall population. The horizontal thick line corresponds to the 95% prediction interval.

**Figure 5:**
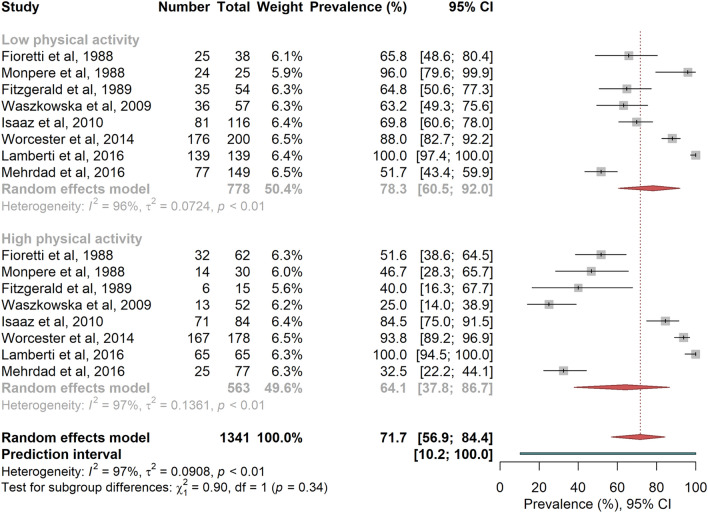
Random-effects meta-analysis of return-to-work prevalence according to occupational physical activity. The squares and horizontal lines correspond to the study-specific prevalence and 95% CIs. Proportionally sized boxes represent the weight of each study. The diamond represents the pooled prevalence and 95% CI of the overall population. The horizontal thick line corresponds to the 95% prediction interval.

RTW overall random effects pooled prevalence was higher among white-collar workers compared to blue-collar workers, and among patients with low OPA compared to those with high OPA (Figs. 4 and 5), even if the differences were not statistically significant ^22,24,26,43,45,50,53,62^ (respectively 81.2% vs. 65.0%, P = 0.251, I^2^ = 96.7%, PI [11.1%; 100%], and 78.3% vs. 64.1%, P = 0.344, I^2^ = 96.7%, PI [10.2%; 100%]; no evidence for small study effects).

Table 1 and Supplementary Fig. S14 online shows RTW prevalence estimates of PCTA patients^26,35,36,42,45,47,51,56,58,60,61^ and CABG patients^24,25,31,34,35,42,50,56,59,60,62^, with respectively 80.8% and 75.9% (P = 0.524, I^2^ = 98.6%, PI [39.7%; 99.7%]). Small study effects were observed in the CABG subgroup (respectively P-values for Begg and Egger tests, 0.64 and 0.01). In the three months following the intervention, CABG patients were less likely to RTW than PCTA patients (40.9% vs. 51.4%, P = 0.347, I^2^ = 0%).

### Sensitivity analyses

As previously stated, heterogeneity in the 0–3 months follow-up group significantly decreased (from I^2^ = 96.6% to I^2^ = 68.1%) when the two larger studies were removed from the model. Similar approaches in the other subgroups made no significant changes on heterogeneity.

Results of RTW prevalence estimates, by type of event, according to the characteristics of the studies and the clinical and socio-professional characteristics of the patients are summarized in Supplementary Table S3 online. RTW prevalence estimates by time of follow-up were similar for all types of events. Regarding acute events, RTW prevalence estimates were higher in patients recruited after 2001 (85.1% versus 73.1%, P = 0.042), in patients with a higher LVEF (73.6% versus 54.7%, P = 0.086) and a higher education level (85.2% versus 74.5%, P = 0.054). RTW prevalence estimates after stable angina or an elective coronary intervention were lower among blue-collars compared to white-collars (35.9% versus 63.0%, P < 0.001) and people with a high occupational physical activity (33.4% versus 57.2%).

## Discussion

This study is to our knowledge the first systematic review of prospective studies about RTW after a coronary event. The objectives were to assess the prevalence of RTW after a coronary event and to identify the determinants which could impact job resumption with particular interest in work conditions.

The main finding obtained from the 43 included articles was a RTW overall random effects pooled prevalence of 81.1% with a progressive increase of RTW prevalence estimates from 47.3% before three months to reach 88.0% at six months, followed by a substantial decrease to obtain a final RTW prevalence estimate at 80%. Prevalence estimates did not vary significantly according to gender, age, study quality, regions, date of recruitment of the participants, methods of evaluation of the disease and outcome, and type of treatment. Few studies focused on the occupational conditions which could impact RTW were published. RTW prevalence estimates among white collars or among people with high educational level tends to be higher compared to blue collars and low education level. In line with these results, the pooled prevalence of RTW among patients with low OPA was estimated at 78.3% versus 64.1% among those with high OPA.

Subgroup meta-analyses according to psychological factors was not done since these factors were seldom considered. Similarly, few studies provided data on RTW according to LVEF, and because of the different time of measurement (baseline, during the acute event or at discharge) and the different thresholds used, results should be carefully interpretated.

### Strengths and weaknesses of the study

A comprehensive systematic research using a broad search strategy was conducted by multidisciplinary specialists in cardiology, epidemiology, and occupational health allowing a better understanding of the types of coronary events as well as the return-to-work conditions. Recall and selection bias which are frequently encountered in cross-sectional and retrospective studies were minimized by including only prospective studies in the present meta-analysis. The large number of analyzed studies covering a wide period and the large number of cases (34,964) allowed to provide robust estimates of prevalence of RTW and to perform subgroup and sensitivity analyses.

However substantial heterogeneity was observed in our study. Indeed, the meta-analyses yielded extreme I^2^ values (about 99%) and wide PI (ranging between 20 and 100%, depending on the model). Although extreme heterogeneity is commonly observed in meta-analyses of prevalence as shown by Migliavaca et al.^64^ (median I^2^ of 134 prevalence meta-analyses assessed at 96.9% [IQR 90.5–98.7]), we sought to examine the potential sources of heterogeneity in our study. Heterogeneity remains in the subgroup and sensitivity analyses. Combining studies differing in several aspects such as the country, date and size of the study, or length of follow-up may partially explain these results. Moreover, some individual characteristics (e.g., cardiovascular risk factors) could not be used in the analyses because they were scarcely reported with the added hurdle due to the varying definitions. The type of coronary event can also be discussed. Most of the included studies considered patients with MI or ACS but few of them involved patients with angina. Moreover, some studies including patients who had undergone PCTA or CABG did not detailed the exact diagnosis. Also, the variability of the definition used for RTW could be involved. Indeed, little information was available regarding the conditions of RTW: part-time or full-time, possibility of workstation changes at the workplace, partial or full recovery of capacity allowing to maintain the employees at their previous workstation. Thus, results should be interpreted with caution.

Unpublished studies and the selection of English and French-written studies can constitute a potential publication bias.

### RTW varied in time after a coronary event

We highlighted that RTW pooled prevalence estimates varied with the time of the follow-up. RTW prevalence estimates varied from 47.3% before three months, 88.0% at 3–6 months, 77% at 6–12 month, to 80% after 12 months. In their systematic review without meta-analysis, Sun et al. reported that RTW occurred in 21.5% to 62.5% of the participants within three months which agrees with the expected prevalences observed in our meta-analyses (PI [26.6; 68.4])^65^. They also suggested that 55.9% to 90% of the participants RTW within 6 to 12 months while our estimations were wider (expected prevalences between 20.2 and 100%). Failure to maintain working can be explained by early retirement, depression but also by the recurrence of a new coronary event. Indeed, in a study based on Swedish registries^66^, the authors have shown that the risk of non-fatal MI, non-fatal stroke, or cardiovascular death after a MI reached 18.3% in the up-coming year. Moreover, depression following a coronary event is frequently described^39^.

### Date of implementation and country of the study

The WHO proposed very early, in 1964, a definition of cardiac rehabilitation followed in the nineties by the first European recommendations focused on patients with heart failure and aimed to improve physical capacity. However, it must be noted that despite of the unquestionable benefit of cardiac rehabilitation, the access to rehabilitation programs is still heterogeneous^67^. Urbinati et al.^4^ have reported that, probably due to the different type of health systems, the difficult access to these programs, and the economic cost, only 35% of the patients with ST-segment elevated MI have undergone this type of care in occidental countries.

Regarding the therapeutic and diagnostic progresses, we hypothesized that the year of the implementation of the studies could influence RTW, but a subgroup analysis revealed no significant difference when comparing prevalence before or after 2000.

Most of the studies took place in Europe (> 70%) and RTW did not differ between the WHO regions.

### Type of treatment during hospitalization

PCTA and CABG have considerably improved patient care and their quality of life. Based on the available studies^24–26,31,34–36,42,45,47,50,51,56,59–62^, our results did not highlight any significant difference in the pooled prevalence estimates of work resumption between these two interventions. However, we can assume that time taken to RTW is longer for patients with CABG than those with PCTA^68^. To explain these contrasted results, we can suppose that other essential determinants of RTW were omitted as factors related to workstation, and notably psychological factors.

### Method and quality of the studies

The methods used to determine the coronary event (use of the ICD from registries, clinician’s expertise, or patient self-report) as well as the way to assess RTW (self-reported or not) did not influence RTW pooled prevalence estimates.

It appears that earlier studies^21–24,27,39,44^ and some more recent^53,55,57^ omitted clinical, demographic or occupational parameters as confounders when assessing RTW.

While the study methodology could be sometimes improved, the global quality of the studies (NOS scale) did not lead to difference in return-to-work prevalence.

### Patients’ expectations

Although data collected for this study were not sufficient to perform analyses on this aspect of RTW, patients’ expectations after a cardiac event appears to be a key element for work resumption. Salzwedel et al.^69^ have shown that after a cardiac event, including MI, patients with initial lower self-assessed occupational prognosis were less likely to be fit to work at the end of cardiac rehabilitation.

### Patients’ sociodemographic characteristics

Despite the results not being significant, RTW pooled prevalence estimates in men seemed higher than in women which was also observed by Sun et al.^65^ As reported by Gauthier et al.^70^ in a study based on the French ischemic heart diseases registries, although medical care improved over time in both men and women, men receive more frequently revascularization treatment, platelet aggregation inhibitors and statins prescription, and functional rehabilitation which could participate in a delayed or unsuccessful RTW in women.

Whereas Sun et al.^65^ systematic review suggested that older participants were less likely to RTW based on seven studies, our meta-analysis did not highlight any significant difference of RTW prevalence estimates between age classes.

### Work conditions

RTW after an ACS depends on medical management, clinical, psychological, and occupational factors. The meta-analysis of the few studies detailing RTW according to socio-professional categories and workload has shown a lower prevalence of work resumption among blue-collar workers and those who perform a job with high physical activity even if no significant difference was observed^22,24,26,43,45,50,53,62^.

No studies aiming to implement modifications of work conditions at the workplace has been carried out. We noted some trials consisting in interventions to assist RTW in patient with coronary heart diseases during the rehabilitation program period. According to Hegewald et al. review and meta-analysis^11^, there is no definitive conclusion on the efficacy of psychological counselling aiming to erase some of the misconceptions and reassure patients about the fears they may have regarding the disease and the perspective to RTW. Also, programs based only on physical exercises did not significantly improve the number of patients returning to work.

### Implications for clinicians and policymakers

Occupational physical constraints seem to have a negative role on RTW while psychological factors at work are insufficiently investigated. A better understanding of the real-life work conditions influencing RTW is crucial since physical and psychological constraints at the workstation, before and after the event, can constitute hurdles to maintain coronary patients in the labor market. A failed RTW could indeed lead to occupational disintegration, negative repercussion in one social life, business disorganization, and more widely to an increased cost for the society.

Thus, the detailed knowledge of work conditions, the collaboration between the occupational health and the health care teams, and an extended and regular support could help to anticipate and ensure an adapted, successful, and permanent RTW.

## Conclusions

This systematic review and meta-analysis of prospective studies have shown an overall random-effects pooled RTW prevalence of 81.1% after a coronary event, in other words a pooled prevalence of no work resumption of nearly 20%. After a gradual increase during the first 6 months following the event (88.0%), this pooled prevalence lessens later (80%). Occupational physical constraints have a negative impact on RTW while psychological factors at work are insufficiently investigated.

Essential cardiovascular determinants of RTW were often omitted. For example, LVEF evolution could be reported more precisely (during the acute event, at discharge, and after cardiac rehabilitation). Given the few studies investigating the stress conditions at the workstation, this occupational factor should be considered in future research to identify patients in need of psychosocial assistance to support them with appropriate interventions. Physical constraints should also be systematically examined in future studies.

Thus, we note the need to implement further studies to determine the occupational conditions which could play a role in earlier work resumption. A better understanding of the whole spectrum of occupational constraints is necessary to coordinate, anticipate and ensure a successful return to work.

## Supplementary Information

Supplementary Figures.

Supplementary Tables.

Supplementary Information 3.

Supplementary Information 4.

## Data Availability

The data underlying this article will be shared on reasonable request to the corresponding author.
