# Supplementary material for: Prevalence and determinants of return to work after various coronary events: meta-analysis of prospective studies
Source: Sci Rep. 2022 Sep 12;12:15348. doi: 10.1038/s41598-022-19467-z (PMC9468005; doi:10.1038/s41598-022-19467-z)

Supplementary Figure S1. NOS scale scores for the studies included in the meta-analysis

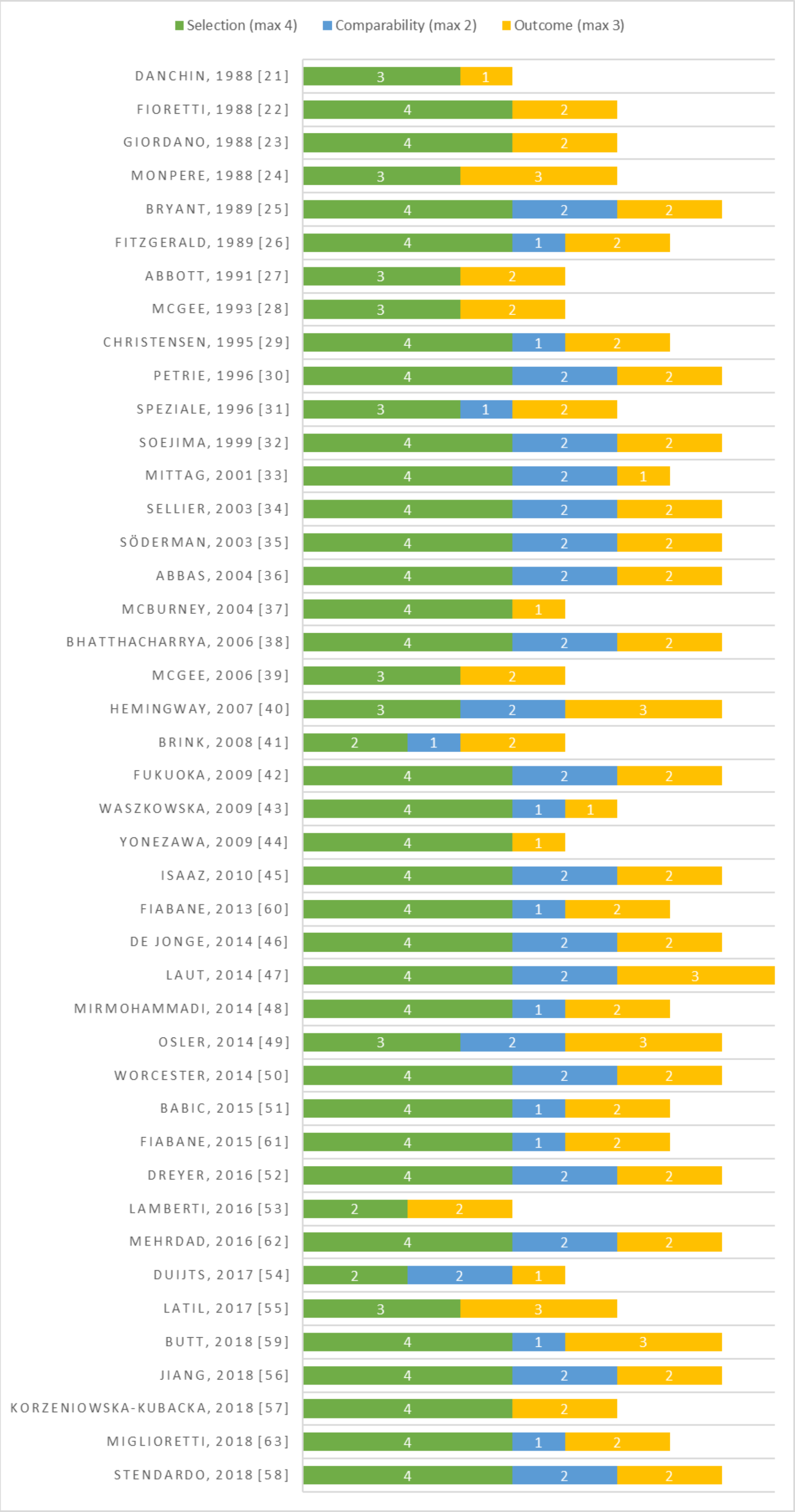

Supplementary Figure S2. Random-effects meta-analysis of return-to-work prevalence considering the longest follow-up. The squares and horizontal lines correspond to the study-specific prevalence and 95% CIs. Proportionally sized boxes represent the weight of each study. The diamond represents the pooled prevalence and 95% CI of the overall population. The horizontal thick line corresponds to the 95% prediction interval

Study	Number	Total	Weight	Prevalence (%)	95% CI
Danchin et al, 1988	26	41	2.1%	63.4	[46.9; 77.9]
Fioretti et al, 1988	58	100	2.3%	58.0	[47.7; 67.8]
Giordano et al, 1988	96	106	2.3%	90.6	[83.3; 95.4]
Monpere et al, 1988	41	56	2.2%	73.2	[59.7; 84.2]
Bryant et al, 1989	42	59	2.2%	71.2	[57.9; 82.2]
Fitzgerald et al, 1989	67	77	2.3%	87.0	[77.4; 93.6]
Abbott et al, 1991	32	57	2.2%	56.1	[42.4; 69.3]
McGee et al, 1993	102	102	2.3%	100.0	[96.4; 100.0]
Christensen et al, 1995	29	62	2.2%	46.8	[34.0; 59.9]
Petrie et al, 1996	60	76	2.3%	78.9	[68.1; 87.5]
Speziale et al, 1996	250	318	2.4%	78.6	[73.7; 83.0]
Soejima et al, 1999	92	111	2.3%	82.9	[74.6; 89.4]
Mittag et al, 2001	74	119	2.3%	62.2	[52.8; 70.9]
Sellier et al, 2003	340	504	2.4%	67.5	[63.2; 71.5]
Soderman et al, 2003	155	198	2.4%	78.3	[71.9; 83.8]
Abbas et al, 2004	353	436	2.4%	81.0	[77.0; 84.5]
McBurney et al, 2004	68	89	2.3%	76.4	[66.2; 84.8]
Bhatthacharrya et al, 2006	101	126	2.3%	80.2	[72.1; 86.7]
McGee et al, 2006	195	363	2.4%	53.7	[48.4; 58.9]
Hemingway et al, 2007	341	341	2.4%	100.0	[98.9; 100.0]
Brink et al, 2008	40	50	2.2%	80.0	[66.3; 90.0]
Fukuoka et al, 2009	186	212	2.4%	87.7	[82.5; 91.8]
Waszkowska et al, 2009	80	168	2.4%	47.6	[39.9; 55.5]
Yonezawa et al, 2009	109	109	2.3%	100.0	[96.7; 100.0]
Isaaz et al, 2010	152	200	2.4%	76.0	[69.5; 81.7]
Fiabane et al, 2013	39	83	2.3%	47.0	[35.9; 58.3]
de Jonge et al, 2014	133	173	2.4%	76.9	[69.9; 82.9]
Laut et al, 2014	3694	4061	2.4%	91.0	[90.0; 91.8]
Mirmohammadi et al, 2014	134	174	2.4%	77.0	[70.0; 83.0]
Osler et al, 2014	10079	15468	2.4%	65.2	[64.4; 65.9]
Worcester et al, 2014	343	378	2.4%	90.7	[87.4; 93.5]
Babic et al, 2015	145	145	2.3%	100.0	[97.5; 100.0]
Fiabane et al, 2015	69	78	2.3%	88.5	[79.2; 94.6]
Dreyer et al, 2016	1457	1680	2.4%	86.7	[85.0; 88.3]
Lamberti et al, 2016	204	204	2.4%	100.0	[98.2; 100.0]
Mehrdad et al, 2016	196	226	2.4%	86.7	[81.6; 90.9]
Duijts et al, 2017	78	191	2.4%	40.8	[33.8; 48.2]
Latil et al, 2017	187	216	2.4%	86.6	[81.3; 90.8]
Butt et al, 2018	5524	6031	2.4%	91.6	[90.9; 92.3]
Jiang et al, 2018	875	1566	2.4%	55.9	[53.4; 58.4]
Korzeniowska-Kubacka et al, 2018	38	38	2.1%	100.0	[90.7; 100.0]
Miglioretti et al, 2018	63	70	2.2%	90.0	[80.5; 95.9]
Stendardo et al, 2018	95	102	2.3%	93.1	[86.4; 97.2]
**Random effects model**		**34964**	**100.0%**	**81.1**	**[75.8; 85.8]**
**Prediction interval**					**[41.2; 100.0]**

Heterogeneity: $I^2 = 99\%$, $\tau^2 = 0.0420$, $p = 0$

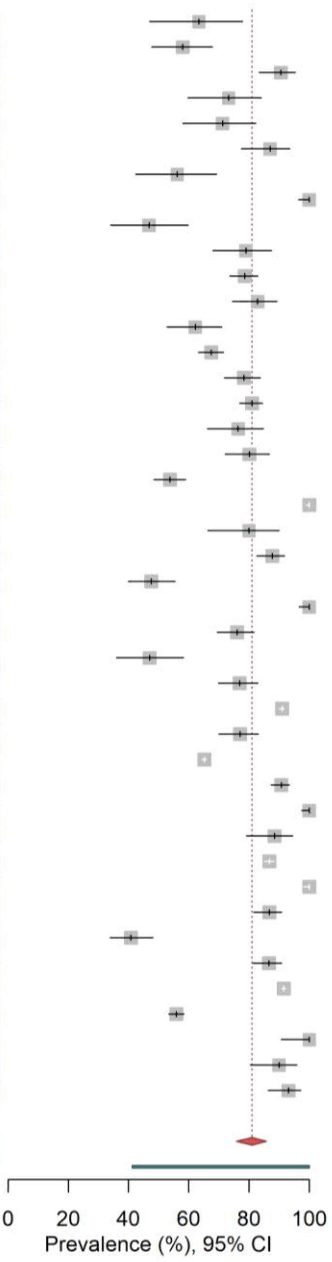

Supplementary Figure S3. Funnel plot, Begg's and Egger's tests to assess the presence of publication bias (considering the longest follow-up for each study)

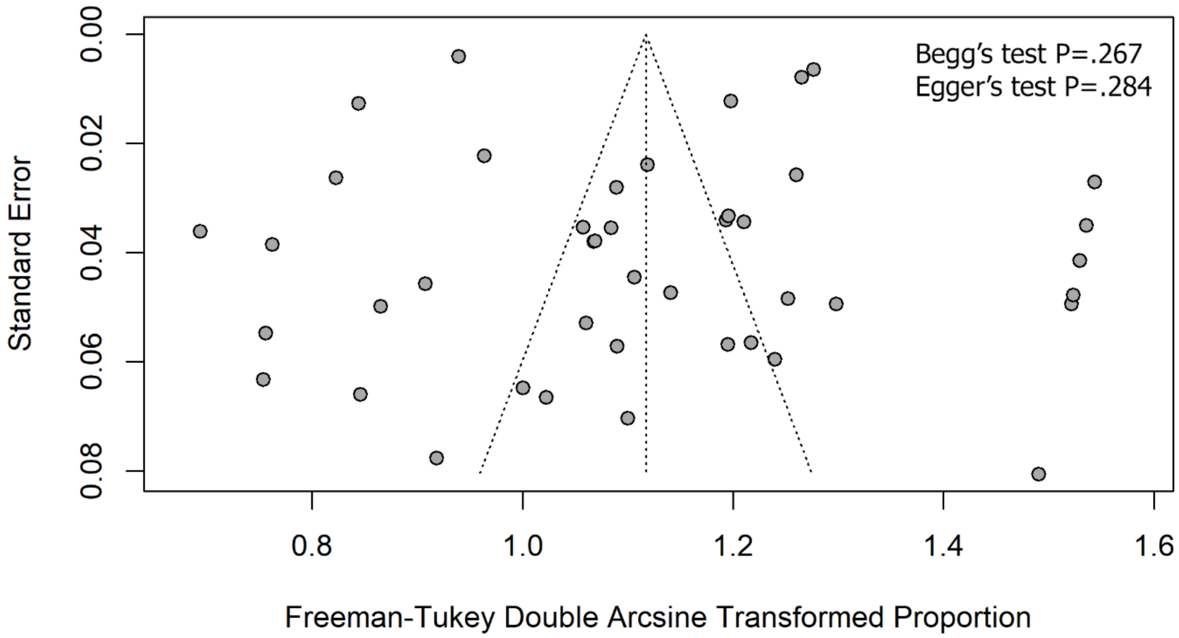

Supplementary Figure S4. Random-effects meta-analysis of return-to-work prevalence according to follow-up time. The squares and horizontal lines correspond to the study-specific prevalence and 95% CIs. Proportionally sized boxes represent the weight of each study. The diamond represents the pooled prevalence and 95% CI of the overall population. The horizontal thick line corresponds to the 95% prediction interval

Study	Number	Total	Weight	Prevalence (%)	95% CI
Follow-up duration =]0 - 3] months					
Fioretti et al, 1988	58	100	1.8%	58.0	[47.7; 67.8]
Bryant et al, 1989	28	70	1.7%	40.0	[28.5; 52.4]
Fitzgerald et al, 1989	41	69	1.7%	59.4	[46.9; 71.1]
Abbott et al, 1991	21	63	1.7%	33.3	[22.0; 46.3]
Abbas et al, 2004	230	450	1.8%	51.1	[46.4; 55.8]
Fiabane et al, 2013	39	83	1.8%	47.0	[35.9; 58.3]
de Jonge et al, 2014	86	186	1.8%	46.2	[38.9; 53.7]
Laut et al, 2014	1109	4061	1.8%	27.3	[25.9; 28.7]
Mirmohammadi et al, 2014	104	174	1.8%	59.8	[52.1; 67.1]
Osler et al, 2014	8150	21926	1.8%	37.2	[36.5; 37.8]
Mehrdad et al, 2016	102	226	1.8%	45.1	[38.5; 51.9]
Latil et al, 2017	123	216	1.8%	56.9	[50.1; 63.6]
Stendardo et al, 2018	60	102	1.8%	58.8	[48.6; 68.5]
Random effects model		27726	23.2%	47.3	[41.7; 52.8]
Prediction interval	.	.	--		[26.6; 68.4]
Heterogeneity: $I^2 = 97\%$, $\tau^2 = 0.0086$, $p < 0.01$					
Follow-up duration =]3 - 6] months					
Giordano et al, 1988	96	106	1.8%	90.6	[83.3; 95.4]
Fitzgerald et al, 1989	67	77	1.8%	87.0	[77.4; 93.6]
Abbott et al, 1991	37	68	1.7%	54.4	[41.9; 66.5]
Petrie et al, 1996	60	76	1.8%	78.9	[68.1; 87.5]
Abbas et al, 2004	353	436	1.8%	81.0	[77.0; 84.5]
Brink et al, 2008	40	50	1.7%	80.0	[66.3; 90.0]
Fukuoka et al, 2009	186	212	1.8%	87.7	[82.5; 91.8]
Yonezawa et al, 2009	109	109	1.8%	100.0	[96.7; 100.0]
Lamberti et al, 2016	204	204	1.8%	100.0	[98.2; 100.0]
Mehrdad et al, 2016	196	226	1.8%	86.7	[81.6; 90.9]
Miglioretti et al, 2018	63	70	1.7%	90.0	[80.5; 95.9]
Random effects model		1634	19.5%	88.0	[79.8; 94.3]
Prediction interval	.	.	--		[50.1; 100.0]
Heterogeneity: $I^2 = 95\%$, $\tau^2 = 0.0321$, $p < 0.01$					
Follow-up duration =]6 - 12] months					
Monpere et al, 1988	41	56	1.7%	73.2	[59.7; 84.2]
Bryant et al, 1989	42	59	1.7%	71.2	[57.9; 82.2]
Abbott et al, 1991	32	57	1.7%	56.1	[42.4; 69.3]
Soejima et al, 1999	92	111	1.8%	82.9	[74.6; 89.4]
Mittag et al, 2001	74	119	1.8%	62.2	[52.8; 70.9]
Sellier et al, 2003	340	504	1.8%	67.5	[63.2; 71.5]
Soderman et al, 2003	155	198	1.8%	78.3	[71.9; 83.8]
McBurney et al, 2004	68	89	1.8%	76.4	[66.2; 84.8]
Bhatthacharrya et al, 2006	101	126	1.8%	80.2	[72.1; 86.7]
McGee et al, 2006	195	363	1.8%	53.7	[48.4; 58.9]
Hemingway et al, 2007	341	341	1.8%	100.0	[98.9; 100.0]
de Jonge et al, 2014	133	173	1.8%	76.9	[69.9; 82.9]
Laut et al, 2014	3473	4061	1.8%	85.5	[84.4; 86.6]
Mirmohammadi et al, 2014	134	174	1.8%	77.0	[70.0; 83.0]
Osler et al, 2014	8777	21869	1.8%	40.1	[39.5; 40.8]
Worcester et al, 2014	343	378	1.8%	90.7	[87.4; 93.5]
Fiabane et al, 2015	69	78	1.8%	88.5	[79.2; 94.6]
Dreyer et al, 2016	1457	1680	1.8%	86.7	[85.0; 88.3]
Latil et al, 2017	187	216	1.8%	86.6	[81.3; 90.8]
Butt et al, 2018	4827	6031	1.8%	80.0	[79.0; 81.0]
Jiang et al, 2018	875	1566	1.8%	55.9	[53.4; 58.4]
Stendardo et al, 2018	95	102	1.8%	93.1	[86.4; 97.2]
Random effects model		38351	39.4%	77.4	[66.6; 86.7]
Prediction interval	.	.	--		[20.2; 100.0]
Heterogeneity: $I^2 = 100\%$, $\tau^2 = 0.0804$, $p = 0$					
Follow-up duration = >12 months					
Danchin et al, 1988	26	41	1.7%	63.4	[46.9; 77.9]
McGee et al, 1993	102	102	1.8%	100.0	[96.4; 100.0]
Christensen et al, 1995	29	62	1.7%	46.8	[34.0; 59.9]
Speziale et al, 1996	250	318	1.8%	78.6	[73.7; 83.0]
Isaaz et al, 2010	152	200	1.8%	76.0	[69.5; 81.7]
Laut et al, 2014	3694	4061	1.8%	91.0	[90.0; 91.8]
Osler et al, 2014	10079	15468	1.8%	65.2	[64.4; 65.9]
Babic et al, 2015	145	145	1.8%	100.0	[97.5; 100.0]
Duijts et al, 2017	78	191	1.8%	40.8	[33.8; 48.2]
Butt et al, 2018	5524	6031	1.8%	91.6	[90.9; 92.3]
Random effects model		26619	17.9%	80.2	[68.0; 90.1]
Prediction interval	.	.	--		[29.3; 100.0]
Heterogeneity: $I^2 = 100\%$, $\tau^2 = 0.0486$, $p = 0$					
**Random effects model**		**94330**	**100.0%**	**74.0**	**[67.6; 79.9]**
**Prediction interval**					**[23.2; 100.0]**
Heterogeneity: $I^2 = 100\%$, $\tau^2 = 0.0683$, $p = 0$					
Test for subgroup differences: $\chi^2_3 = 72.51$, df = 3 ($p < 0.01$)					

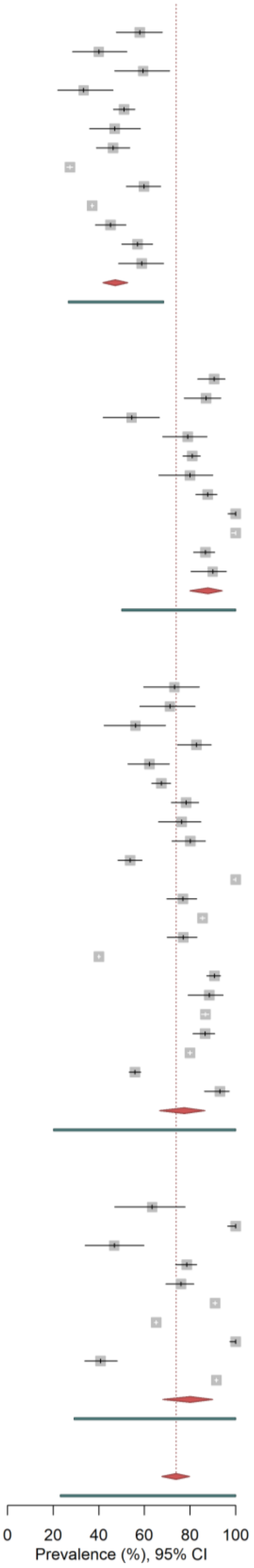

Supplementary Figure S5. Random-effects meta-analysis of return-to-work prevalence according to recruitment date. The squares and horizontal lines correspond to the study-specific prevalence and 95% CIs. Proportionally sized boxes represent the weight of each study. The diamond represents the pooled prevalence and 95% CI of the overall population. The horizontal thick line corresponds to the 95% prediction interval

Study	Number	Total	Weight	Prevalence (%)	95% CI
<2001					
Danchin et al, 1988	26	41	2.1%	63.4	[46.9; 77.9]
Fioretti et al, 1988	58	100	2.3%	58.0	[47.7; 67.8]
Giordano et al, 1988	96	106	2.3%	90.6	[83.3; 95.4]
Monpere et al, 1988	41	56	2.2%	73.2	[59.7; 84.2]
Bryant et al, 1989	42	59	2.2%	71.2	[57.9; 82.2]
Fitzgerald et al, 1989	67	77	2.3%	87.0	[77.4; 93.6]
Abbott et al, 1991	32	57	2.2%	56.1	[42.4; 69.3]
McGee et al, 1993	102	102	2.3%	100.0	[96.4; 100.0]
Christensen et al, 1995	29	62	2.2%	46.8	[34.0; 59.9]
Petrie et al, 1996	60	76	2.3%	78.9	[68.1; 87.5]
Speziale et al, 1996	250	318	2.4%	78.6	[73.7; 83.0]
Soejima et al, 1999	92	111	2.3%	82.9	[74.6; 89.4]
Mittag et al, 2001	74	119	2.3%	62.2	[52.8; 70.9]
Sellier et al, 2003	340	504	2.4%	67.5	[63.2; 71.5]
Soderman et al, 2003	155	198	2.4%	78.3	[71.9; 83.8]
Abbas et al, 2004	353	436	2.4%	81.0	[77.0; 84.5]
McBurney et al, 2004	68	89	2.3%	76.4	[66.2; 84.8]
Hemingway et al, 2007	341	341	2.4%	100.0	[98.9; 100.0]
Isaaz et al, 2010	152	200	2.4%	76.0	[69.5; 81.7]
de Jonge et al, 2014	133	173	2.4%	76.9	[69.9; 82.9]
Laut et al, 2014	3694	4061	2.4%	91.0	[90.0; 91.8]
Butt et al, 2018	5524	6031	2.4%	91.6	[90.9; 92.3]
Random effects model		13317	50.9%	79.6	[74.4; 84.4]
Heterogeneity: $I^2 = 97\%$, $\tau^2 = 0.0194$, $p < 0.01$					
>=2001					
Bhatthacharrya et al, 2006	101	126	2.3%	80.2	[72.1; 86.7]
McGee et al, 2006	195	363	2.4%	53.7	[48.4; 58.9]
Brink et al, 2008	40	50	2.2%	80.0	[66.3; 90.0]
Fukuoka et al, 2009	186	212	2.4%	87.7	[82.5; 91.8]
Waszkowska et al, 2009	80	168	2.4%	47.6	[39.9; 55.5]
Yonezawa et al, 2009	109	109	2.3%	100.0	[96.7; 100.0]
Fiabane et al, 2013	39	83	2.3%	47.0	[35.9; 58.3]
Mirmohammadi et al, 2014	134	174	2.4%	77.0	[70.0; 83.0]
Osler et al, 2014	10079	15468	2.4%	65.2	[64.4; 65.9]
Worcester et al, 2014	343	378	2.4%	90.7	[87.4; 93.5]
Babic et al, 2015	145	145	2.3%	100.0	[97.5; 100.0]
Fiabane et al, 2015	69	78	2.3%	88.5	[79.2; 94.6]
Dreyer et al, 2016	1457	1680	2.4%	86.7	[85.0; 88.3]
Lamberti et al, 2016	204	204	2.4%	100.0	[98.2; 100.0]
Mehrdad et al, 2016	196	226	2.4%	86.7	[81.6; 90.9]
Duijts et al, 2017	78	191	2.4%	40.8	[33.8; 48.2]
Latil et al, 2017	187	216	2.4%	86.6	[81.3; 90.8]
Jiang et al, 2018	875	1566	2.4%	55.9	[53.4; 58.4]
Korzeniowska-Kubacka et al, 2018	38	38	2.1%	100.0	[90.7; 100.0]
Miglioretti et al, 2018	63	70	2.2%	90.0	[80.5; 95.9]
Stendardo et al, 2018	95	102	2.3%	93.1	[86.4; 97.2]
Random effects model		21647	49.1%	82.7	[75.7; 88.8]
Heterogeneity: $I^2 = 99\%$, $\tau^2 = 0.0387$, $p = 0$					
**Random effects model**		**34964**	**100.0%**	**81.1**	**[75.8; 85.8]**
**Prediction interval**					**[41.2; 100.0]**
Heterogeneity: $I^2 = 99\%$, $\tau^2 = 0.0420$, $p = 0$					
Test for subgroup differences: $\chi^2_1 = 0.55$, df = 1 ($p = 0.46$)					

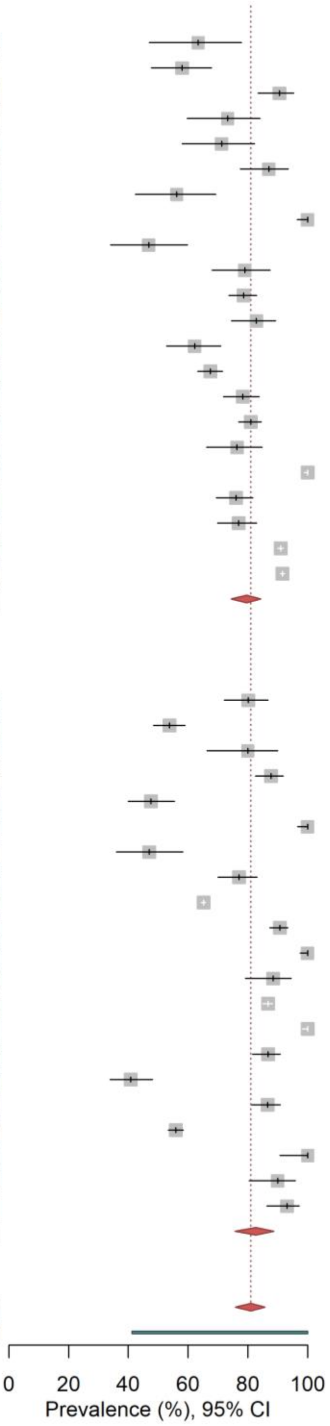

Supplementary Figure S6. Random-effects meta-analysis of return-to-work prevalence according to disease definition. The squares and horizontal lines correspond to the study-specific prevalence and 95% CIs. Proportionally sized boxes represent the weight of each study. The diamond represents the pooled prevalence and 95% CI of the overall population. The horizontal thick line corresponds to the 95% prediction interval

Study	Number	Total	Weight	Prevalence (%)	95% CI
Clinical					
Danchin et al, 1988	26	41	2.1%	63.4	[46.9; 77.9]
Fioretti et al, 1988	58	100	2.3%	58.0	[47.7; 67.8]
Giordano et al, 1988	96	106	2.3%	90.6	[83.3; 95.4]
Monpere et al, 1988	41	56	2.2%	73.2	[59.7; 84.2]
Bryant et al, 1989	42	59	2.2%	71.2	[57.9; 82.2]
Fitzgerald et al, 1989	67	77	2.3%	87.0	[77.4; 93.6]
Abbott et al, 1991	32	57	2.2%	56.1	[42.4; 69.3]
McGee et al, 1993	102	102	2.3%	100.0	[96.4; 100.0]
Christensen et al, 1995	29	62	2.2%	46.8	[34.0; 59.9]
Petrie et al, 1996	60	76	2.3%	78.9	[68.1; 87.5]
Speziale et al, 1996	250	318	2.4%	78.6	[73.7; 83.0]
Soejima et al, 1999	92	111	2.3%	82.9	[74.6; 89.4]
Mittag et al, 2001	74	119	2.3%	62.2	[52.8; 70.9]
Sellier et al, 2003	340	504	2.4%	67.5	[63.2; 71.5]
Abbas et al, 2004	353	436	2.4%	81.0	[77.0; 84.5]
Bhatthacharrya et al, 2006	101	126	2.3%	80.2	[72.1; 86.7]
McGee et al, 2006	195	363	2.4%	53.7	[48.4; 58.9]
Brink et al, 2008	40	50	2.2%	80.0	[66.3; 90.0]
Fukuoka et al, 2009	186	212	2.4%	87.7	[82.5; 91.8]
Waszkowska et al, 2009	80	168	2.4%	47.6	[39.9; 55.5]
Yonezawa et al, 2009	109	109	2.3%	100.0	[96.7; 100.0]
Isaaz et al, 2010	152	200	2.4%	76.0	[69.5; 81.7]
Fiabane et al, 2013	39	83	2.3%	47.0	[35.9; 58.3]
de Jonge et al, 2014	133	173	2.4%	76.9	[69.9; 82.9]
Laut et al, 2014	3694	4061	2.4%	91.0	[90.0; 91.8]
Mirmohammadi et al, 2014	134	174	2.4%	77.0	[70.0; 83.0]
Worcester et al, 2014	343	378	2.4%	90.7	[87.4; 93.5]
Babic et al, 2015	145	145	2.3%	100.0	[97.5; 100.0]
Fiabane et al, 2015	69	78	2.3%	88.5	[79.2; 94.6]
Dreyer et al, 2016	1457	1680	2.4%	86.7	[85.0; 88.3]
Lamberti et al, 2016	204	204	2.4%	100.0	[98.2; 100.0]
Mehrdad et al, 2016	196	226	2.4%	86.7	[81.6; 90.9]
Latil et al, 2017	187	216	2.4%	86.6	[81.3; 90.8]
Jiang et al, 2018	875	1566	2.4%	55.9	[53.4; 58.4]
Korzeniowska-Kubacka et al, 2018	38	38	2.1%	100.0	[90.7; 100.0]
Miglioretti et al, 2018	63	70	2.2%	90.0	[80.5; 95.9]
Stendardo et al, 2018	95	102	2.3%	93.1	[86.4; 97.2]
**Random effects model**		**12646**	**85.7%**	**81.3**	**[75.8; 86.2]**
Heterogeneity: I^2 = 98%, τ^2 = 0.0393, p = 0					
Interview					
Hemingway et al, 2007	341	341	2.4%	100.0	[98.9; 100.0]
Duijts et al, 2017	78	191	2.4%	40.8	[33.8; 48.2]
**Random effects model**		**532**	**4.8%**	**81.1**	**[7.8; 100.0]**
Heterogeneity: I^2 = 100%, τ^2 = 0.3602, p < 0.01					
ICD					
Soderman et al, 2003	155	198	2.4%	78.3	[71.9; 83.8]
McBurney et al, 2004	68	89	2.3%	76.4	[66.2; 84.8]
Osler et al, 2014	10079	15468	2.4%	65.2	[64.4; 65.9]
Butt et al, 2018	5524	6031	2.4%	91.6	[90.9; 92.3]
**Random effects model**		**21786**	**9.5%**	**78.8**	**[57.5; 94.0]**
Heterogeneity: I^2 = 100%, τ^2 = 0.0540, p = 0					
**Random effects model**		**34964**	**100.0%**	**81.1**	**[75.8; 85.8]**
**Prediction interval**					**[41.2; 100.0]**
Heterogeneity: I^2 = 99%, τ^2 = 0.0420, p = 0					
Test for subgroup differences: χ^2_2 = 0.06, df = 2 (p = 0.97)					

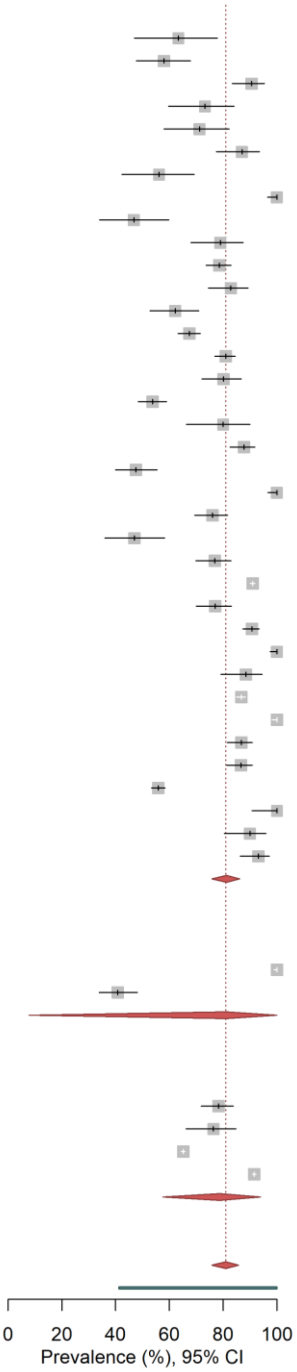

Supplementary Figure S7. Random-effects meta-analysis of return-to-work prevalence according to outcome measure method. The squares and horizontal lines correspond to the study-specific prevalence and 95% CIs. Proportionally sized boxes represent the weight of each study. The diamond represents the pooled prevalence and 95% CI of the overall population. The horizontal thick line corresponds to the 95% prediction interval

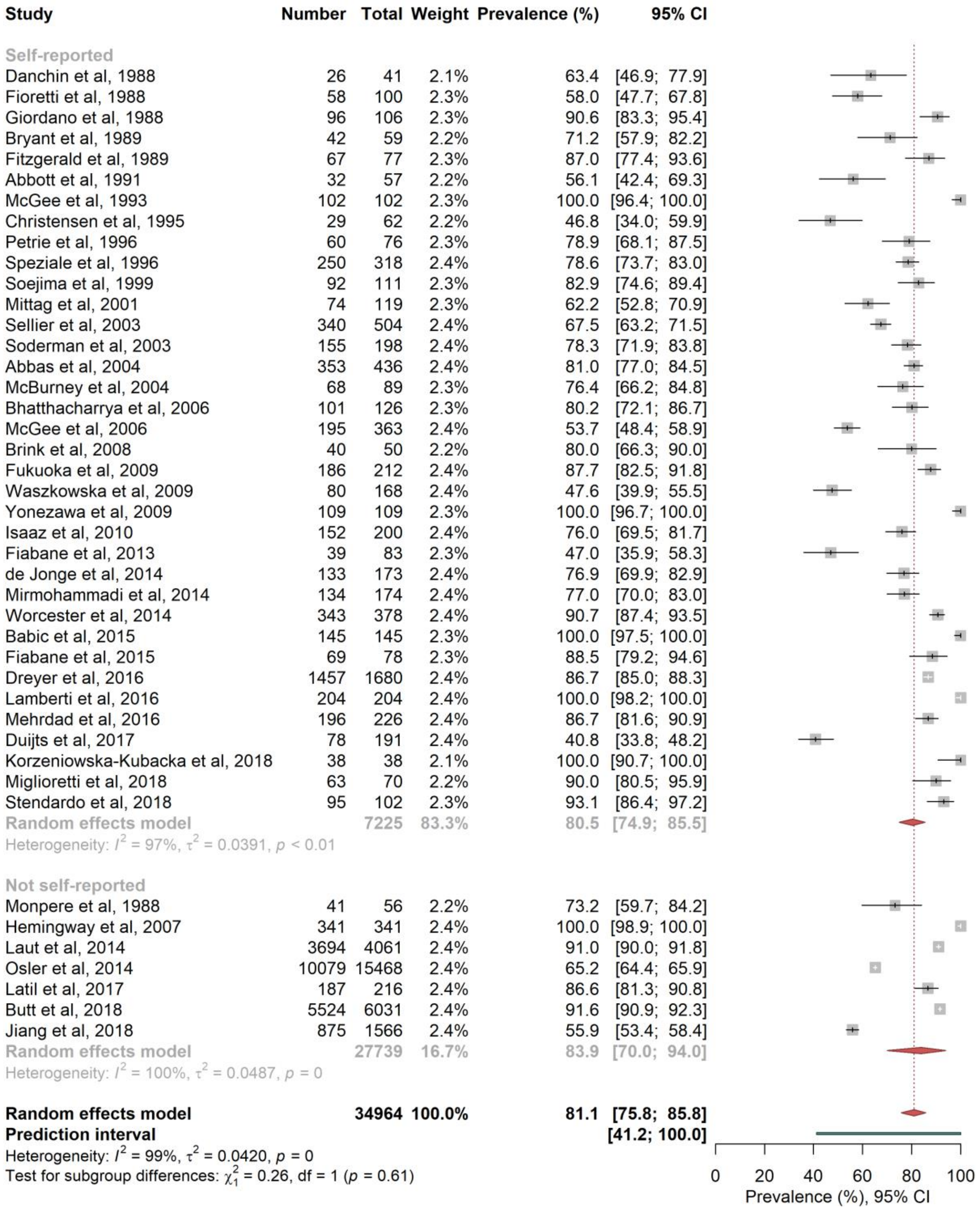

Supplementary Figure S8. Random-effects meta-analysis of return-to-work prevalence according to study quality. The squares and horizontal lines correspond to the study-specific prevalence and 95% CIs. Proportionally sized boxes represent the weight of each study. The diamond represents the pooled prevalence and 95% CI of the overall population. The horizontal thick line corresponds to the 95% prediction interval

Study	Number	Total	Weight	Prevalence (%)	95% CI
**7 stars or more**					
Bryant et al, 1989	42	59	2.2%	71.2	[57.9; 82.2]
Fitzgerald et al, 1989	67	77	2.3%	87.0	[77.4; 93.6]
Christensen et al, 1995	29	62	2.2%	46.8	[34.0; 59.9]
Petrie et al, 1996	60	76	2.3%	78.9	[68.1; 87.5]
Soejima et al, 1999	92	111	2.3%	82.9	[74.6; 89.4]
Mittag et al, 2001	74	119	2.3%	62.2	[52.8; 70.9]
Sellier et al, 2003	340	504	2.4%	67.5	[63.2; 71.5]
Soderman et al, 2003	155	198	2.4%	78.3	[71.9; 83.8]
Abbas et al, 2004	353	436	2.4%	81.0	[77.0; 84.5]
Bhatthacharrya et al, 2006	101	126	2.3%	80.2	[72.1; 86.7]
Hemingway et al, 2007	341	341	2.4%	100.0	[98.9; 100.0]
Fukuoka et al, 2009	186	212	2.4%	87.7	[82.5; 91.8]
Isaaz et al, 2010	152	200	2.4%	76.0	[69.5; 81.7]
Fiabane et al, 2013	39	83	2.3%	47.0	[35.9; 58.3]
de Jonge et al, 2014	133	173	2.4%	76.9	[69.9; 82.9]
Laut et al, 2014	3694	4061	2.4%	91.0	[90.0; 91.8]
Mirmohammadi et al, 2014	134	174	2.4%	77.0	[70.0; 83.0]
Osler et al, 2014	10079	15468	2.4%	65.2	[64.4; 65.9]
Worcester et al, 2014	343	378	2.4%	90.7	[87.4; 93.5]
Babic et al, 2015	145	145	2.3%	100.0	[97.5; 100.0]
Fiabane et al, 2015	69	78	2.3%	88.5	[79.2; 94.6]
Dreyer et al, 2016	1457	1680	2.4%	86.7	[85.0; 88.3]
Mehrdad et al, 2016	196	226	2.4%	86.7	[81.6; 90.9]
Butt et al, 2018	5524	6031	2.4%	91.6	[90.9; 92.3]
Jiang et al, 2018	875	1566	2.4%	55.9	[53.4; 58.4]
Miglioretti et al, 2018	63	70	2.2%	90.0	[80.5; 95.9]
Stendardo et al, 2018	95	102	2.3%	93.1	[86.4; 97.2]
Random effects model		32756	63.4%	81.6	[75.2; 87.2]
Heterogeneity: $I^2 = 99\%$, $\tau^2 = 0.0397$, $p = 0$					
**<7 stars**					
Danchin et al, 1988	26	41	2.1%	63.4	[46.9; 77.9]
Fioretti et al, 1988	58	100	2.3%	58.0	[47.7; 67.8]
Giordano et al, 1988	96	106	2.3%	90.6	[83.3; 95.4]
Monpere et al, 1988	41	56	2.2%	73.2	[59.7; 84.2]
Abbott et al, 1991	32	57	2.2%	56.1	[42.4; 69.3]
McGee et al, 1993	102	102	2.3%	100.0	[96.4; 100.0]
Speziale et al, 1996	250	318	2.4%	78.6	[73.7; 83.0]
McBurney et al, 2004	68	89	2.3%	76.4	[66.2; 84.8]
McGee et al, 2006	195	363	2.4%	53.7	[48.4; 58.9]
Brink et al, 2008	40	50	2.2%	80.0	[66.3; 90.0]
Waszkowska et al, 2009	80	168	2.4%	47.6	[39.9; 55.5]
Yonezawa et al, 2009	109	109	2.3%	100.0	[96.7; 100.0]
Lamberti et al, 2016	204	204	2.4%	100.0	[98.2; 100.0]
Duijts et al, 2017	78	191	2.4%	40.8	[33.8; 48.2]
Latil et al, 2017	187	216	2.4%	86.6	[81.3; 90.8]
Korzeniowska-Kubacka et al, 2018	38	38	2.1%	100.0	[90.7; 100.0]
Random effects model		2208	36.6%	80.2	[67.3; 90.5]
Heterogeneity: $I^2 = 98\%$, $\tau^2 = 0.0852$, $p < 0.01$					
**Random effects model**		**34964**	**100.0%**	**81.1**	**[75.8; 85.8]**
**Prediction interval**					**[41.2; 100.0]**
Heterogeneity: $I^2 = 99\%$, $\tau^2 = 0.0420$, $p = 0$					
Test for subgroup differences: $\chi^2_1 = 0.05$, df = 1 ($p = 0.82$)					

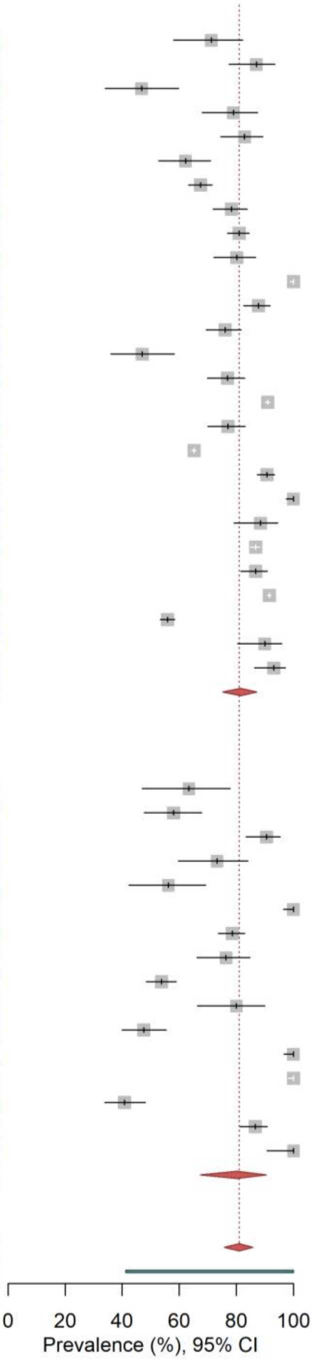

Supplementary Figure S9. Random-effects meta-analysis of return-to-work prevalence according to study location. The squares and horizontal lines correspond to the study-specific prevalence and 95% CIs. Proportionally sized boxes represent the weight of each study. The diamond represents the pooled prevalence and 95% CI of the overall population. The horizontal thick line corresponds to the 95% prediction interval

Study	Number	Total	Weight	Prevalence (%)	95% CI
International					
Abbas et al, 2004	353	436	2.4%	81.0	[77.0; 84.5]
Fukuoka et al, 2009	186	212	2.4%	87.7	[82.5; 91.8]
Dreyer et al, 2016	1457	1680	2.4%	86.7	[85.0; 88.3]
Random effects model		2328	7.2%	85.2	[81.1; 88.9]
Heterogeneity: $I^2 = 79\%$, $\tau^2 = 0.0018$, $p < 0.01$					
Europe					
Danchin et al, 1988	26	41	2.1%	63.4	[46.9; 77.9]
Fioretti et al, 1988	58	100	2.3%	58.0	[47.7; 67.8]
Giordano et al, 1988	96	106	2.3%	90.6	[83.3; 95.4]
Monpere et al, 1988	41	56	2.2%	73.2	[59.7; 84.2]
Bryant et al, 1989	42	59	2.2%	71.2	[57.9; 82.2]
Abbott et al, 1991	32	57	2.2%	56.1	[42.4; 69.3]
McGee et al, 1993	102	102	2.3%	100.0	[96.4; 100.0]
Christensen et al, 1995	29	62	2.2%	46.8	[34.0; 59.9]
Speziale et al, 1996	250	318	2.4%	78.6	[73.7; 83.0]
Mittag et al, 2001	74	119	2.3%	62.2	[52.8; 70.9]
Sellier et al, 2003	340	504	2.4%	67.5	[63.2; 71.5]
Soderman et al, 2003	155	198	2.4%	78.3	[71.9; 83.8]
Bhatthacharrya et al, 2006	101	126	2.3%	80.2	[72.1; 86.7]
McGee et al, 2006	195	363	2.4%	53.7	[48.4; 58.9]
Hemingway et al, 2007	341	341	2.4%	100.0	[98.9; 100.0]
Brink et al, 2008	40	50	2.2%	80.0	[66.3; 90.0]
Waszkowska et al, 2009	80	168	2.4%	47.6	[39.9; 55.5]
Isaaz et al, 2010	152	200	2.4%	76.0	[69.5; 81.7]
Fiabane et al, 2013	39	83	2.3%	47.0	[35.9; 58.3]
de Jonge et al, 2014	133	173	2.4%	76.9	[69.9; 82.9]
Laut et al, 2014	3694	4061	2.4%	91.0	[90.0; 91.8]
Osler et al, 2014	10079	15468	2.4%	65.2	[64.4; 65.9]
Babic et al, 2015	145	145	2.3%	100.0	[97.5; 100.0]
Fiabane et al, 2015	69	78	2.3%	88.5	[79.2; 94.6]
Lamberti et al, 2016	204	204	2.4%	100.0	[98.2; 100.0]
Duijts et al, 2017	78	191	2.4%	40.8	[33.8; 48.2]
Latil et al, 2017	187	216	2.4%	86.6	[81.3; 90.8]
Butt et al, 2018	5524	6031	2.4%	91.6	[90.9; 92.3]
Korzeniowska-Kubacka et al, 2018	38	38	2.1%	100.0	[90.7; 100.0]
Miglioretti et al, 2018	63	70	2.2%	90.0	[80.5; 95.9]
Stendardo et al, 2018	95	102	2.3%	93.1	[86.4; 97.2]
Random effects model		29830	71.8%	79.8	[73.0; 85.8]
Heterogeneity: $I^2 = 99\%$, $\tau^2 = 0.0480$, $p = 0$					
America					
Fitzgerald et al, 1989	67	77	2.3%	87.0	[77.4; 93.6]
McBurney et al, 2004	68	89	2.3%	76.4	[66.2; 84.8]
Random effects model		166	4.6%	81.9	[70.5; 91.0]
Heterogeneity: $I^2 = 67\%$, $\tau^2 = 0.0061$, $p = 0.08$					
Western Pacific					
Petrie et al, 1996	60	76	2.3%	78.9	[68.1; 87.5]
Soejima et al, 1999	92	111	2.3%	82.9	[74.6; 89.4]
Yonezawa et al, 2009	109	109	2.3%	100.0	[96.7; 100.0]
Worcester et al, 2014	343	378	2.4%	90.7	[87.4; 93.5]
Jiang et al, 2018	875	1566	2.4%	55.9	[53.4; 58.4]
Random effects model		2240	11.7%	85.0	[62.3; 98.4]
Heterogeneity: $I^2 = 99\%$, $\tau^2 = 0.0872$, $p < 0.01$					
Eastern Mediterranean					
Mirmohammadi et al, 2014	134	174	2.4%	77.0	[70.0; 83.0]
Mehrdad et al, 2016	196	226	2.4%	86.7	[81.6; 90.9]
Random effects model		400	4.7%	82.2	[71.8; 90.7]
Heterogeneity: $I^2 = 84\%$, $\tau^2 = 0.0067$, $p = 0.01$					
**Random effects model**		**34964**	**100.0%**	**81.1**	**[75.8; 85.8]**
**Prediction interval**					**[41.2; 100.0]**
Heterogeneity: $I^2 = 99\%$, $\tau^2 = 0.0420$, $p = 0$					
Test for subgroup differences: $\chi^2_4 = 2.45$, df = 4 ($p = 0.65$)					

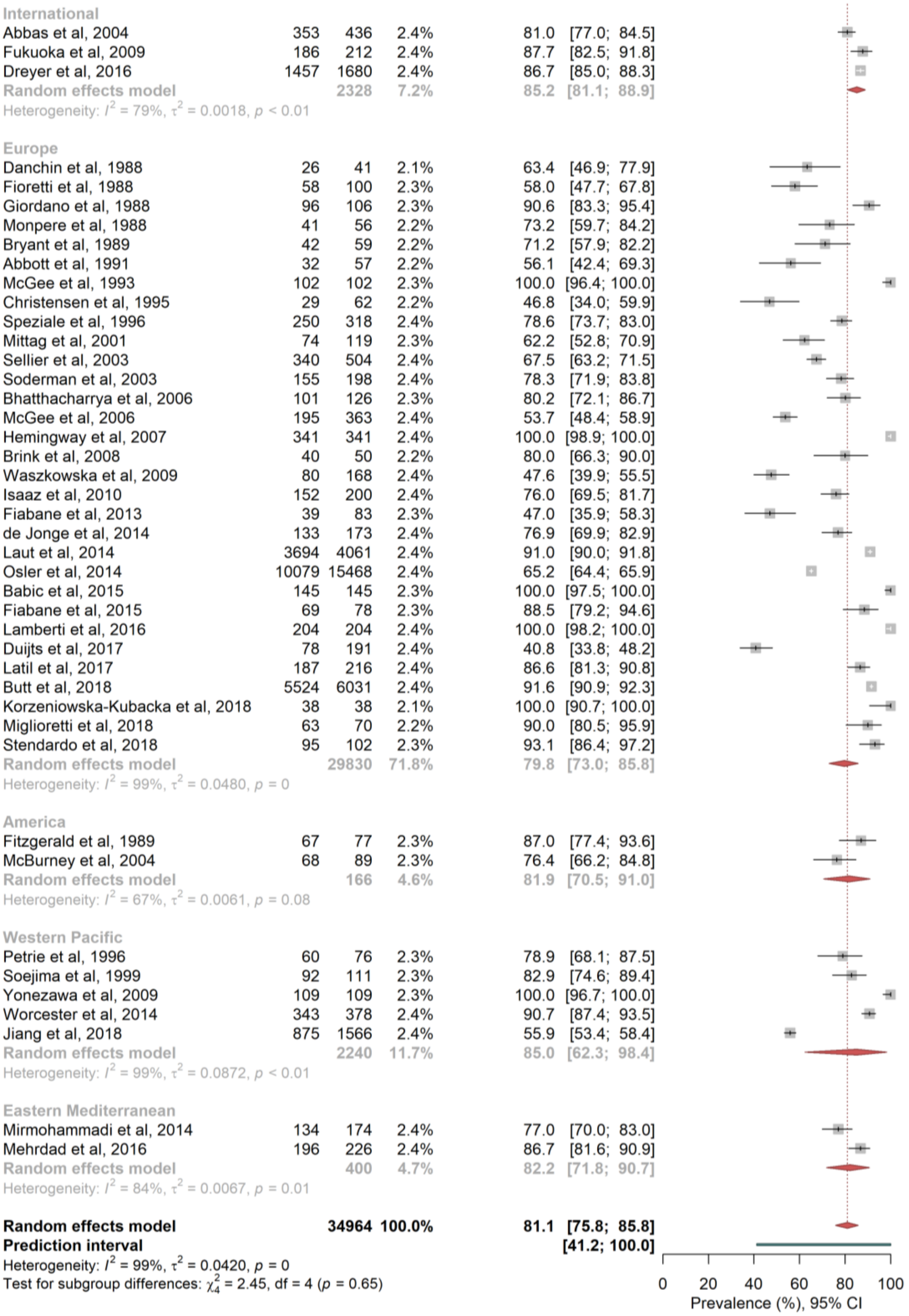

Supplementary Figure S10. Random-effects meta-analysis of return-to-work prevalence according to gender. The squares and horizontal lines correspond to the study-specific prevalence and 95% CIs. Proportionally sized boxes represent the weight of each study. The diamond represents the pooled prevalence and 95% CI of the overall population. The horizontal thick line corresponds to the 95% prediction interval

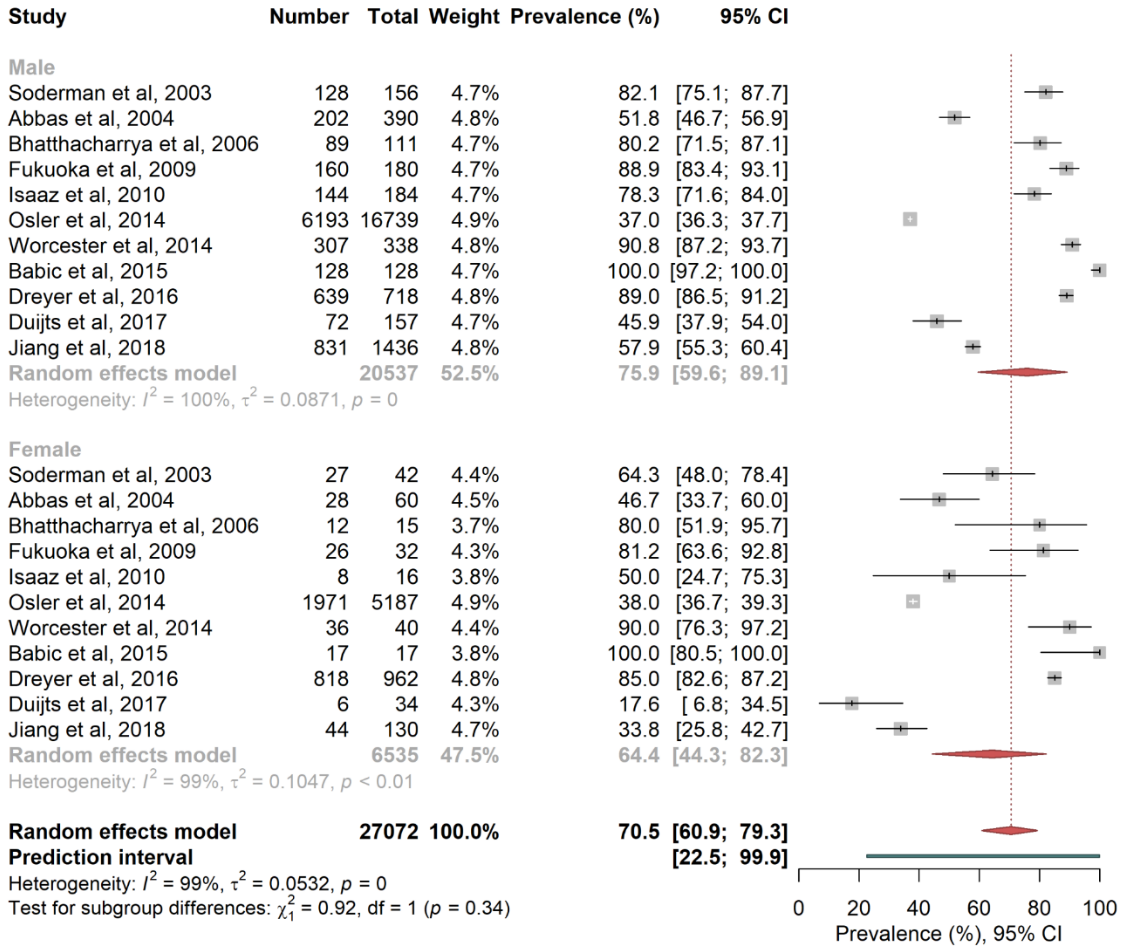

Supplementary Figure S11. Random-effects meta-analysis of return-to-work prevalence according to age. The squares and horizontal lines correspond to the study-specific prevalence and 95% CIs. Proportionally sized boxes represent the weight of each study. The diamond represents the pooled prevalence and 95% CI of the overall population. The horizontal thick line corresponds to the 95% prediction interval

Study	Number	Total	Weight	Prevalence (%)	95% CI
<51 years					
Giordano et al, 1988	96	106	4.5%	90.6	[83.3; 95.4]
Monpere et al, 1988	41	56	4.3%	73.2	[59.7; 84.2]
Sellier et al, 2003	340	504	4.7%	67.5	[63.2; 71.5]
Isaaz et al, 2010	152	200	4.6%	76.0	[69.5; 81.7]
Fiabane et al, 2013	39	83	4.4%	47.0	[35.9; 58.3]
Dreyer et al, 2016	1457	1680	4.7%	86.7	[85.0; 88.3]
Random effects model		2629	27.1%	74.9	[62.8; 85.4]
Heterogeneity: $I^2 = 97\%$, $\tau^2 = 0.0246$, $p < 0.01$					
51-53 years					
Petrie et al, 1996	60	76	4.4%	78.9	[68.1; 87.5]
Mittag et al, 2001	74	119	4.5%	62.2	[52.8; 70.9]
Soderman et al, 2003	155	198	4.6%	78.3	[71.9; 83.8]
Hemingway et al, 2007	341	341	4.6%	100.0	[98.9; 100.0]
Fukuoka et al, 2009	186	212	4.6%	87.7	[82.5; 91.8]
Waszkowska et al, 2009	80	168	4.6%	47.6	[39.9; 55.5]
Lamberti et al, 2016	204	204	4.6%	100.0	[98.2; 100.0]
Latil et al, 2017	187	216	4.6%	86.6	[81.3; 90.8]
Jiang et al, 2018	875	1566	4.7%	55.9	[53.4; 58.4]
Random effects model		3100	41.1%	82.0	[63.5; 95.0]
Heterogeneity: $I^2 = 99\%$, $\tau^2 = 0.1006$, $p < 0.01$					
54 years and more					
Soejima et al, 1999	92	111	4.5%	82.9	[74.6; 89.4]
McBurney et al, 2004	68	89	4.4%	76.4	[66.2; 84.8]
McGee et al, 2006	195	363	4.6%	53.7	[48.4; 58.9]
Yonezawa et al, 2009	109	109	4.5%	100.0	[96.7; 100.0]
Mehrdad et al, 2016	196	226	4.6%	86.7	[81.6; 90.9]
Butt et al, 2018	5524	6031	4.7%	91.6	[90.9; 92.3]
Stendardo et al, 2018	95	102	4.5%	93.1	[86.4; 97.2]
Random effects model		7031	31.8%	86.2	[73.3; 95.4]
Heterogeneity: $I^2 = 98\%$, $\tau^2 = 0.0452$, $p < 0.01$					
**Random effects model**		**12760**	**100.0%**	**81.6**	**[73.9; 88.2]**
**Prediction interval**					**[37.6; 100.0]**

Heterogeneity: $I^2 = 99\%$, $\tau^2 = 0.0472$, $p = 0$

Test for subgroup differences: $\chi^2_2 = 1.90$, df = 2 ($p = 0.39$)

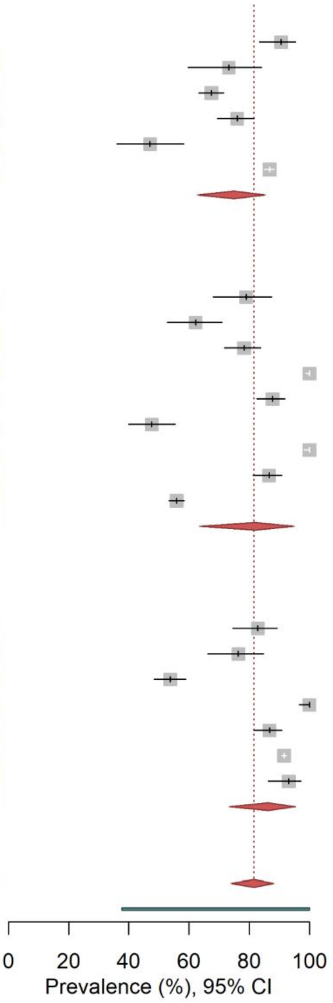

Supplementary Figure S12. Random-effects meta-analysis of return-to-work prevalence according to left ventricular ejection fraction (LVEF). The squares and horizontal lines correspond to the study-specific prevalence and 95% CIs. Proportionally sized boxes represent the weight of each study. The diamond represents the pooled prevalence and 95% CI of the overall population. The horizontal thick line corresponds to the 95% prediction interval

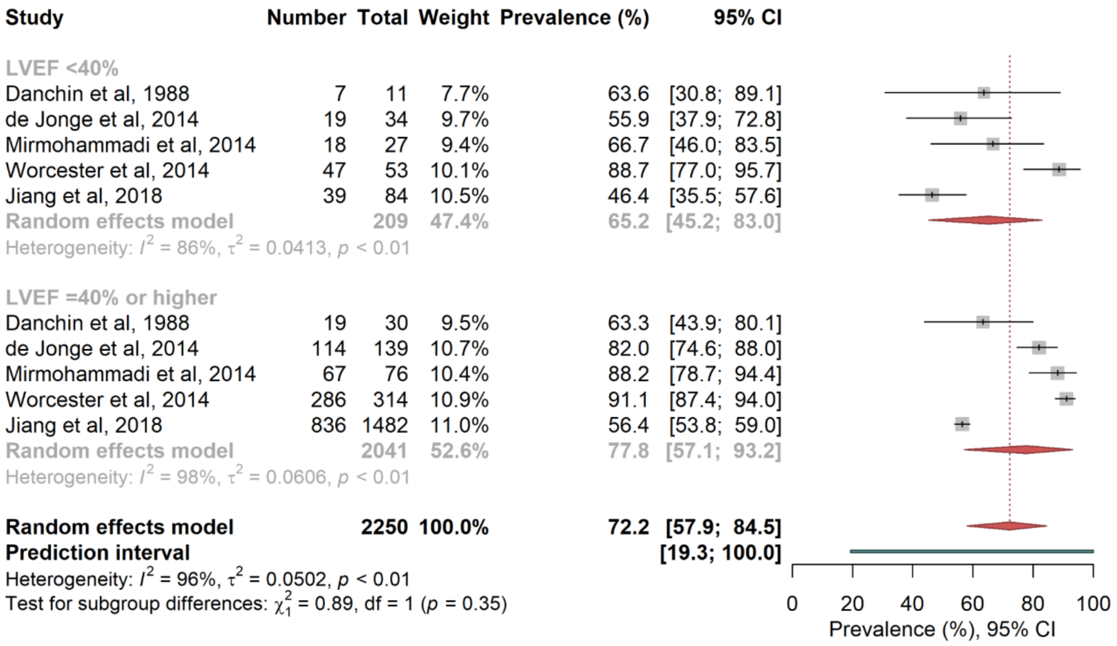

Supplementary Figure S13. Random-effects meta-analysis of return-to-work prevalence according to education level. The squares and horizontal lines correspond to the study-specific prevalence and 95% CIs. Proportionally sized boxes represent the weight of each study. The diamond represents the pooled prevalence and 95% CI of the overall population. The horizontal thick line corresponds to the 95% prediction interval

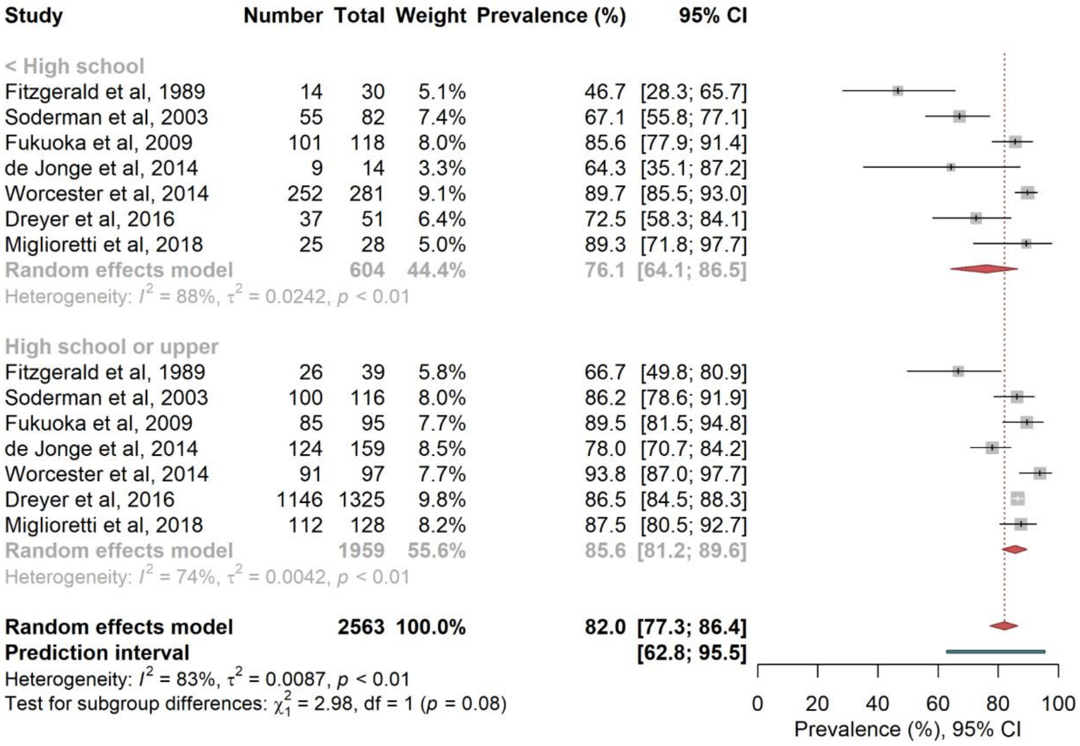

Supplementary Figure S14. Random-effects meta-analysis of return-to-work prevalence according to treatment. The squares and horizontal lines correspond to the study-specific prevalence and 95% CIs. Proportionally sized boxes represent the weight of each study. The diamond represents the pooled prevalence and 95% CI of the overall population. The horizontal thick line corresponds to the 95% prediction interval. CABG, coronary artery bypass graft surgery; PCTA, percutaneous coronary transluminal angioplasty

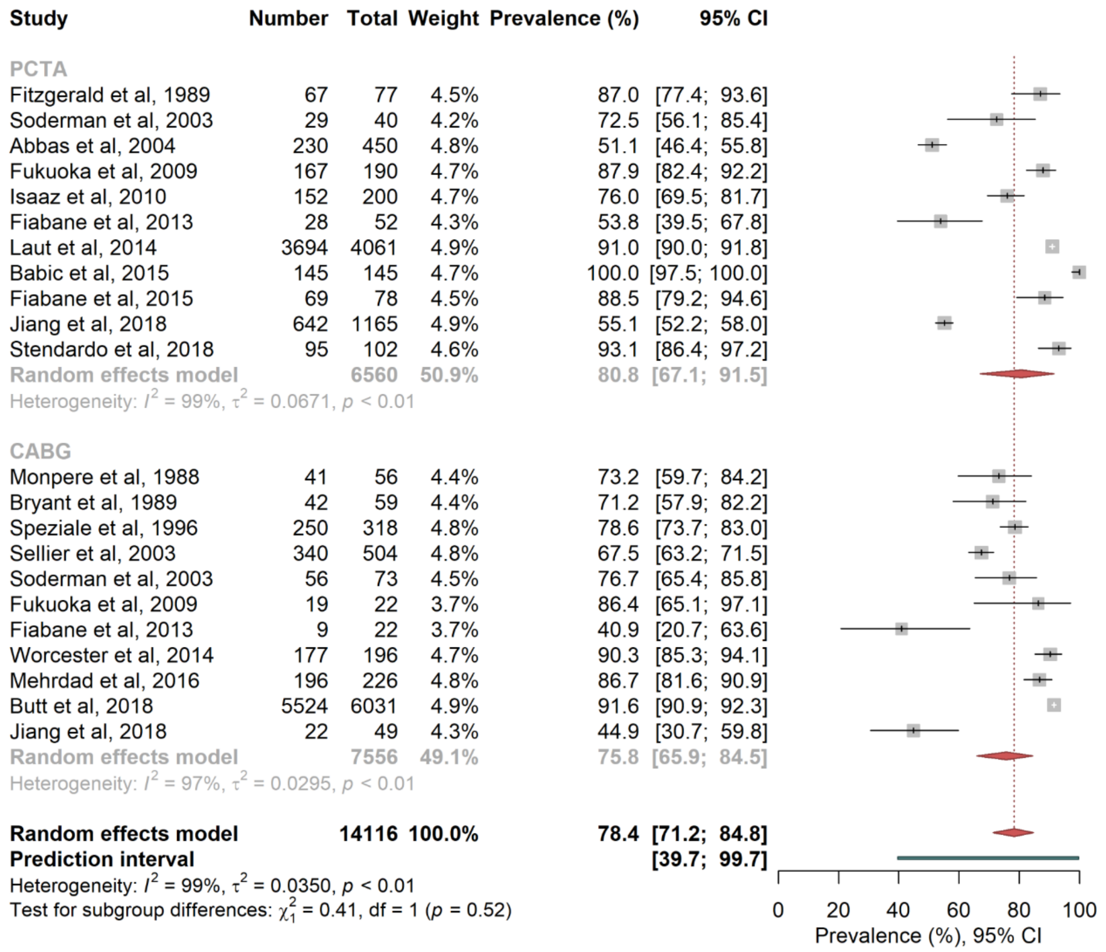

Supplement: Supplementary file 1 — Supplementary Figures. [file 41598_2022_19467_MOESM1_ESM.pdf]
