# Supplementary material for: Prevalence and determinants of return to work after various coronary events: meta-analysis of prospective studies
Source: Sci Rep. 2022 Sep 12;12:15348. doi: 10.1038/s41598-022-19467-z (PMC9468005; doi:10.1038/s41598-022-19467-z)
Supplement: Supplementary file 2 — Supplementary Tables. [file 41598_2022_19467_MOESM2_ESM.pdf]

Supplementary Table S1. Description of the studies included in the meta-analysis

Study	Country, recruitment site and period	Study size (n) and % of men	Follow-up, days[a]	Age, years[b]	Coronary event	Disease definition	RTW evaluation	Number of RTW
**Danchin, 1988 [20]**	France; Nancy university hospital, Cardiology department; 1985	41 (100)	1095.75	NA	MI	clinical	self-reported	26
**Fioretti, 1988 [21]**	The Netherlands; Rotterdam, coronary care unit, thorax center; 1981-1983	100 (NA)	90	NA	MI	clinical	self-reported	58
**Giordano, 1988 [22]**	Italy; hospital; 1987	106 (100)	117 (51)	49 (8)	MI	clinical	self-reported	96
**Monpere, 1988 [23]**	France ; Tours, rehabilitation center 'Bois Gibert'; 1987	57 (98.2)	210 (NA)	50.7 (NA)	CABG	clinical	not self-report	41
**Bryant, 1989 [24]**	England; Oxford cardiac department; 1986	59 (100)	365	NA	Angina, surgery	clinical	self-reported	42
**Fitzgerald, 1989 [25]**	USA; Baltimore, Johns Hopkins Hospital	77 (NA)	180	NA	PTCA	clinical	self-reported	67
**Abbott, 1991 [26]**	England; Royal Preston Hospital, Coronary Care Unit; 1989	57 (100)	365	NA	MI	clinical	self-reported	32
**McGee, 1993 [27]**	Ireland; Dublin, National Cardiothoracic Unit; 1989	102 (NA)	540	NA	PCTA, CABG	clinical	self-reported	102
**Christensen, 1995 [28]**	Denmark; Aalborg Hospital, Department of Cardiology; 1988-1989	62 (83.9)	540	NA	MI	clinical	self-reported	29
**Petrie, 1996 [29]**	New Zealand; Auckland and North Shore Hospitals; 1993-?	76 (93.4)	180	53.1 (7.2)	MI	clinical	self-reported	60
**Speziale, 1996 [30]**	Italy; Rome, Institute of Cardiac Surgery, University "La Sapienza"; 1992	318 (NA)	1140 (180)	NA	Angina, CABG	clinical	self-reported	250
**Soejima, 1999 [31]**	Japan; Kagoshima City area hospitals; 1992-1996	111 (100)	240 (NA)	54.3 (NA)	MI	clinical	self-reported	92
**Mittag, 2001 [32]**	Germany; two hospitals in northern Germany; 1999	119 (0)	365.25	52.3 (5.67)	MI, CABG	clinical	self-reported	74

**Sellier, 2003 [33]**	France; Cardiac rehabilitation departments; 1998-1999	504 (94.8)	365.25	50.5 (NA)	CABG	clinical	self-reported	340
**Söderman, 2003 [34]**	Sweden; Local social insurance agencies; 1992-1994	198 (78.8)	365.25	51.34 (5.3)	MI, CABG, PTCA	ICD	self-reported	155
**Abbas, 2004 [35]**	International; Stent Primary Angioplasty in Myocardial Infarction study; hospitals; 1999	436 (NA)	180	NA	PCTA (after MI)	clinical	self-reported	353
**McBurney, 2004 [36]**	USA; Midwestern academic health system; 1999-2000	89 (80.9)	225 (45)	55.5 (10)	MI	ICD	self-reported	68
**Bhatthacharrya, 2006 [37]**	England; London hospitals; 2001-2004	126 (88.1)	390	NA	ACS	clinical	self-reported	101
**McGee, 2006 [38]**	Ireland; 39 centers with intensive/coronary care unit; 2003	363 (NA)	360	63 ± 12	ACS	clinical	self-reported	195
**Hemingway, 2007 [39]**	Finland; 21 public hospitals; 2000-2002	341 (37)	360	52.7 (NA)	Angina	interview	not self-report (computerized sickness absence records)	341
**Brink, 2008 [40]**	Sweden; rural hospital, Coronary care unit; 2005-2006	50 (NA)	180	NA	MI	clinical	self-reported	40
**Fukuoka, 2009 [41]**	USA, Japan; hospital; 2004-2006	212 (84.9)	180	53 (NA)	ACS	clinical	self-reported	186
**Waszkowska, 2009 [42]**	Poland; Clinical Department of Cardiology, Medical University of Lodz; 2009	168 (100)	NA	53.7 (NA)	MI	clinical	self-reported	80
**Yonezawa, 2009 [43]**	Japan; Cardiovascular center of Kitasato University hospital; 2003-2006	109 (82.6)	180	57 (7)	MI	clinical	self-reported	109
**Isaaz, 2010 [44]**	France; Cardiology department North hospital, CHU de Saint-Étienne; 2000-2004	200 (92)	1260 (NA)	48 (7)	ACS, PCTA	clinical	self-reported	152
**Fiabane, 2013 [59]**	Italy; hospital; 2006-2010	83 (94)	60	49.3 (8.04)	PCTA, CABG	clinical	self-reported	39

**de Jonge, 2014 [45]**	Netherlands; hospitals; 1997-2000	173 (90.8)	365.25	NA	MI	clinical	self-reported	133
**Laut, 2014 [46]**	Denmark; Aalborg, Gentofte, Odense, Rigshospitalet, and Aarhus University Hospital in Skejby; 1999-2011	4061 (84.6)	1461	NA	ACS, PCTA	clinical	not self-reported (National Register on Public Transfer Payments (DREAM))	3694
**Mirmohammadi, 2014 [47]**	Iran; hospital; 2007-2010	174 (NA)	365.25	NA	MI	clinical	self-reported	134
**Osler, 2014 [48]**	Denmark; National Patient Register; 2001-2009	15468 (NA)	1826.25	NA	ACS	ICD	not self-reported (National Register on Public Transfer Payments (DREAM))	10079
**Worcester, 2014 [49]**	Australia; Cardiothoracic Surgical Unit of the Royal Melbourne hospital or Melbourne private hospital; 2008-2010	378 (89.4)	365.25	NA	ACS, CABG	clinical	self-reported	343
**Babic, 2015 [50]**	Croatia; Zagreb Hospital Coronary Care Unit; 2008-2011	145 (88.3)	836.12 (241.63)	NA	ACS, PCTA	clinical	self-reported	145
**Fiabane, 2015 [60]**	Italy; Research and Care Delivery Institution for multidisciplinary Cardiac Rehabilitation; 2006-2011	78 (80.8)	365.25	NA	PCTA	clinical	self-reported	69
**Dreyer, 2016 [51]**	USA, Spain, Australia; VIRGO study, hospitals; 2008-2012	1680 (42.7)	365.25	48 (8)	MI	clinical	self-reported	1457
**Lamberti, 2016 [52]**	Italy; Naples hospital; 2014	204 (97.5)	180	51 (8)	ACS	clinical	self-reported	204
**Mehrdad, 2016 [61]**	Iran; hospital; 2013-2014	226 (99.1)	180	54.2 (8.93)	CABG	clinical	self-reported	196
**Duijts, 2017 [53]**	England; General population; 2002-2010	191 (82.2)	730.5	NA	MI	interview	self-reported	78
**Latil, 2017 [54]**	France; hospital; 2011-2012	216 (97.2)	365.25	53.5 (7)	ACS	clinical	not self-reported	187
**Butt, 2018 [58]**	Denmark; Nationwide administrative registries; 1998-2011	6031 (88.9)	730.5	55 (47, 63)	CABG	procedure codes	not self-reported	5524

**Jiang, 2018 [55]**	China; hospital; 2013-2014	1566 (91.7)	365.25	52.2 (NA)	MI	clinical	not self-reported	875
**Korzeniowska-Kubacka, 2018 [56]**	Poland; hospital; 2017	38 (63.2)	NA	NA	MI	clinical	self-reported	38
**Miglioretti, 2018 [62]**	Italy; hospital; 2013-2014	70 (NA)	180	NA	PCTA, CABG	clinical	self-reported	63
**Stendardo, 2018 [57]**	Italy; Department of Cardiology of the University-Hospital of Ferrara; 2015-2016	102 (88.2)	365.25	56 (50, 60)	MI	clinical	self-reported	95

Table 1 Legend : ACS, acute coronary syndrome; CABG, coronary artery bypass graft surgery; ICD, International Classification of Diseases; MI, myocardial infarction; NA, not available; PCTA, percutaneous coronary transluminal angioplasty; RTW, return to work.

[a]Longest follow-up in days (maximum or mean (standard deviation)); [b]Age in years (mean (standard deviation), median (interquartile range))

Supplementary Table S2. Studies settings

	n (%)
**Regions**	
America	2 (4.7)
Europe	31 (72.1)
Eastern Mediterranean	2 (4.7)
Western Pacific	5 (11.6)
International	3 (7.0)
**Setting**	
Hospital	39 (90.7)
Population register	3 (7.0)
Insurance database	1 (2.3)

Supplementary Table S3. Return to work prevalence, by type of events, according to the characteristics of the studies and the clinical and socio-professional characteristics of the patients

		Acute event					Stable angina				
		**No.**	**RTW %**	**[95%-CI]**	**I²**	**Pᵃ**	**No.**	**RTW %**	**[95%-CI]**	**I²**	**Pᵃ**
**All studies (longest follow-up)**		26	80.3%	[73.6; 86.2]	99.0		13	83.6%	[73.7; 91.6]	98.3	
**Follow-up**	]0 - 3] months	9	47.1%	[40.6; 53.7]	97.5	< 0.001	4	47.4%	[40.5; 54.4]	49.1	< 0.001
	]3 - 6] months	7	87.5%	[73.9; 96.7]	96.8		4	88.2%	[85.1; 91.0]	0.0	
	]6 - 12] months	13	74.4%	[59.1; 87.2]	99.8		5	84.9%	[69.5; 95.6]	98.7	
	>12 months	6	78.2%	[60.7; 91.6]	99.7		4	83.0%	[60.0; 97.4]	99.1	
**Recruitment date**	<2001	11	73.1%	[63.6; 81.6]	96.1	0.042	8	89.2%	[79.5; 96.1]	98.1	0.142
	≥2001	15	85.1%	[77.1; 91.6]	99.0		5	72.8%	[48.4; 91.6]	97.5	
**Disease definition**	Clinical	24	81.1%	[73.8; 87.4]	98.5	0.081	10	83.1%	[73.5; 90.9]	95.1	0.073
	ICD	2	69.9%	[58.7; 80.0]	80.7		1	91.6%	[90.9; 92.3]	-	
	Interview	0	-	-	-		2	81.1%	[7.8; 100.0]	99.7	
**Outcome definition**	Self-reported	22	81.1%	[73.6; 87.6]	97.0	0.612	11	79.8%	[68.3; 89.3]	96.6	0.039
	Not self-reported	4	76.1%	[57.2; 90.8]	99.8		2	97.5%	[83.1; 100]	98.9	
**NOS-scale score**	7 stars or more	15	80.6%	[72.3; 87.9]	99.3	0.912	9	84.1%	[73.3; 92.6]	98.0	0.913
	<7 stars	11	79.9%	[62.8; 92.7]	98.0		4	82.6%	[53.6; 99.0]	98.5	
**WHO region**	International	3	85.2%	[81.1; 88.9]	78.6	0.127	0	-	-	-	0.782
	Europe	17	79.0%	[69.3; 87.3]	99.2		11	83.0%	[71.2; 92.2]	98.6	
	Western Pacific	4	83.4%	[54.3; 99.3]	98.7		0	-	-	-	
	America	1	76.4%	[67.0; 84.7]	-		1	87.0%	[78.5; 93.7]	-	

	Eastern Mediterranean	1	77.0%	[70.4; 83.0]	-		1	86.7%	[82.0; 90.9]	-	
**Gender**	Male	8	76.3%	[57.6; 90.8]	99.6	0.478	1	45.9%	[38.1; 53.7]	-	0.002
	Female	8	66.5%	[42.6; 86.8]	99.2		1	17.7%	[6.4; 32.5]	-	
**Age**	<51 years	2	82.0%	[70.3; 91.3]	92.9	0.944	3	70.2%	[47.7; 88.5]	95.9	<0.001
	51-53 years	6	80.0%	[58.1; 95.2]	99.0		1	100.0%	[99.5; 100]	-	
	54 years and more	5	84.9%	[61.6; 98.5]	98.0		2	89.7%	[84.4; 94.0]	82.5	
**Education level**	< High school	4	74.5%	[62.2; 85.2]	74.0	0.054	2	70.0%	[24.7; 99.5]	92.1	0.699
	≥ High school	4	85.2%	[80.8; 89.1]	64.0		2	78.8%	[55.4; 95.3]	87.2	
**Socio professional category**	White-collar	4	79.5%	[48.1; 98.6]	97.3	0.719	2	63.0%	[54.6; 71.0]	0.0	
	Blue-collar	4	71.2%	[31.6; 97.9]	97.7		2	35.9%	[28.4; 43.8]	0.0	
**Occupational physical activity**	Low	4	79.5%	[48.1; 98.6]	97.3	0.719	2	57.2%	[44.4; 69.5]	63.5	0.005
	High	4	71.2%	[31.6; 97.9]	97.7		2	33.4%	[23.8; 43.6]	0.0	
**Left Ventricular Ejection Fraction**	<40%	4	54.7%	[44.7; 64.4]	23.9	0.086	0	-	-	-	-
	≥40%	4	73.6%	[53.9; 89.4]	95.9		0	-	-	-	
**Treatment**	PCTA	7	82.8%	[65.3; 95.0]	99.4	0.419	3	78.3%	[56.4; 94.2]	91.3	0.839
	CABG	2	66.3%	[23.8; 97.5]	91.5		6	75.8%	[62.1; 87.3]	98.1	

CABG, coronary artery bypass graft surgery; CI, confidence interval; ICD, International Classification of Diseases; N°, number of studies; NOS, Newcastle-Ottawa scale; P, p-value; PCTA, percutaneous coronary transluminal angioplasty; RTW %, return to work prevalence.

[a]Between group difference
